# Strengthening literature search strategies for systematic reviews reporting population health in the Middle East and North Africa: A meta‐research study

**DOI:** 10.1111/jebm.12394

**Published:** 2020-05-24

**Authors:** Karima Chaabna, Sohaila Cheema, Amit Abraham, Ravinder Mamtani

**Affiliations:** ^1^ Institute for Population Health, Weill Cornell Medicine Education City‐Qatar Foundation Doha Qatar

**Keywords:** Africa, gray literature, Middle East, research design, systematic reviews

## Abstract

**Objective:**

In the Middle East and North Africa (MENA), data are produced in languages other than English and available through gray literature sources. We assessed the comprehensiveness of literature search strategies of systematic reviews (SRs) reporting population health primary data on MENA.

**Methods:**

Utilizing the registered protocol (PROSPERO CRD42017076736), we conducted a meta‐research analysis on a cohort of SRs (systematic PubMed search: from 2008 to 2016) and evaluated their search strategies following AMSTAR recommendations.

**Results:**

A total of 379 SRs were included. Few SRs (10.3%, *n *= 39) conducted a comprehensive literature search including at least two databases, reference lists of included primary studies, gray literature sources, and no language restriction. Nevertheless, 90.5% (*n *= 343) searched at least two databases and 67.0% (*n *= 254) searched gray literature sources. Authors from MENA searched statistically more for gray literature than authors from Western countries (*P *= 0.022). Reference lists of the included studies were searched in 40.4% (*n *= 153) of the SRs. Searching the reference lists was positively associated with searching for gray literature (*P *< 0.001). Only 38.8% (*n *= 147) of the SRs had no language restriction or searched in English and in at least one language relevant to MENA, whereas 27.2% (*n *= 103) did not report this information.

**Conclusions:**

Literature searches for SRs reporting population health data on MENA were limited in reporting quality, language restrictions, and lack of reference list searches. This was probably due to lack of adherence to the reporting guidelines. To ensure compilation of optimum evidence, expanding literature searches to reference list search and for additional languages relevant to MENA are required.

## 1 INTRODUCTION

Generating and augmenting knowledge on health problems is essential at regional and global levels for developing health promotion and prevention strategies to tackle these issues through informed, evidence‐based decision making.^1^ Researchers, policymakers, and clinicians should be well informed about population health issues in their countries to find context‐appropriate solutions. This emphasizes the importance of conducting an appropriate literature search to compile the published knowledge on health issues. Well‐conducted systematic reviews (SRs) are commonly recognized as offering the best evidence for informed best practice because they critically appraise and synthesize the available evidence. Thus, they are different from narrative reviews which do not follow a rigorous methodology, potentially leading to biased conclusions and recommendations.^2^, ^3^ Additionally, it is recommended that SRs should be registered in the PROSPERO registry for author accountability, so they follow protocols developed a priori.^4^ Conducting a systematic literature search is a crucial step in any SR that ultimately determines its quality. According to AMSTAR – a critical appraisal tool for SRs that include randomized or nonrandomized studies ^5^, ^6^ – to provide the best evidence a SR must use a comprehensive literature search strategy combining no language restriction, a search of at least two databases relevant to the research question and the reference lists of included studies, and a search for gray literature.

Gray literature are materials which are produced at all levels of government, academics, business, and industry in print and electronic formats but are not controlled by commercial publishers.^7^ Although non‐English gray literature may not affect the results of SRs for interventional studies (clinical field),^8^ this is probably not the case for SRs reporting population health studies conducted in a region such as the Middle East and North Africa (MENA). English is not an official language of any of the MENA countries, and many have Arabic and/or French as the medium of instruction in universities and colleges.^9^ A substantial proportion of the epidemiological research in MENA is likely to be disseminated in languages other than English, and most probably via channels not controlled by commercial publishers, such as in non‐indexed journals affiliated to local universities. This is a potential challenge for a comprehensive literature search, since the information may not be easy to search and/or to retrieve.

Our meta‐research study is part of the Population Health Publications Assessment Project aiming to assess the methodological quality and the use of gray literature in published SRs on population health in MENA.^10^, ^11^ This study aimed to evaluate the comprehensiveness of the literature search strategies utilized in SRs reporting population health data in MENA and to explore the variability of the search strategies, and to provide recommendations to strengthen literature search strategies for SRs.

## 2 METHODS

### 2.1 Study design

Based on a published protocol^9^ (PROSPERO registration number CRD42017076736),^12^ we conducted a meta‐research analysis of the comprehensiveness of the systematic reviews' literature search. First, we conducted a systematic overview to systematically identify a cohort of SRs. The study is reported following PRISMA statements.

### 2.2 Eligibility criteria and selection

We included all SRs reporting population health data on countries in the MENA region, published and indexed on PubMed(13) between 2008 and 2016. The 20 countries included in our review were Algeria, Bahrain, Djibouti, Egypt, Iraq, Jordan, Kuwait, Lebanon, Libya, Morocco, Oman, Pakistan, Palestine, Qatar, Saudi Arabia, Sudan, Syria, Tunisia, United Arab Emirates, and Yemen. The selection process of these 20 MENA countries was described in our protocol.^9^

We included SRs of observational studies presenting descriptive epidemiological data and excluded clinical and interventional study SRs. Narrative reviews or synthesis papers analyzing primary studies, which did not follow a systematic process were excluded. Based on PRISMA‐P terminology,^14^ we defined a SR as a review of primary studies reporting a search strategy for at least one electronic database along with eligibility criteria, which were applied during a multistage process of study selection. Population health was defined as the health outcomes of a group of individuals including the distribution of such outcomes within the group.^15^

The literature search strategy published in our protocol^9^ was built using MeSH terms and Title/Abstract terms covering the 20 MENA countries, inclusive of their populations (supplementary material 1). Similar search criteria have been utilized in SRs for population health in MENA.^11^, ^16^, ^17^, ^18^ A double title/abstract and full‐text screening were conducted independently by two authors.

### 2.3 Data extraction

Systematic review characteristics (gray literature search, reference list checking, database search, language restriction, year of publication, geographical coverage, and health topic) and authors' country of affiliations and potential collaborations were extracted by one author and checked for accuracy by a second author. Discrepancy meetings including all the authors were set up after each stage of the screening process and following data extraction checks. We retrieved the journals' impact factor (JIF) during the year of publication from the Institute of Scientific Information's Journal Citation Report.^19^ The list of included SRs along with their characteristics are presented in Supplementary Material S2.

### 2.4 Data analysis

We assessed the comprehensiveness of the literature search strategy used in the included SRs by focusing on four criteria: searches of at least two databases and reference list, search for gray literature, and use of languages during the literature search, as recommended by AMSTAR guidelines.^6^ We also evaluated the proportion of SRs reporting the search period of their literature search as recommended by the original version of AMSTAR checklist(6). The literature source “contacting an expert in the particular field of study”^6^ is considered as a possible strategy while searching for gray literature. The search of clinical trial registries was not considered relevant to SRs included in our overview and hence, was not included in our assessment criteria.

Analyses were conducted based on institutional author affiliation as per the following categories: (1) “Inside”—all authors affiliated to institutions located in MENA and/or neighboring countries, (2) “Outside”—all authors affiliated to institutions located in non‐MENA and non‐neighboring (NMNN) countries, and (3) “Collaboration”—authors affiliated to institutions located in NMNN countries collaborating with authors from MENA and/or neighboring countries. Authors were categorized as belonging to the MENA countries if one of their institutional affiliations was in one of the 20 selected countries and to neighboring countries if their institutional affiliations were in a country not included in the 20 selected MENA countries but in the Middle East, South Asia, or Africa. Due to the geographic proximity of the neighboring countries, which often share socio‐economic and cultural aspects with some of the 20 selected countries, we differentiated these authors affiliated to institutions located in these countries from authors from NMNN countries located, for instance, in Europe, Australia, or the American continent. The reasoning behind this is that a few of the neighboring countries such as Iran^20^, ^21^, ^22^, ^23^, Turkey,^23^ and Somalia^21^, ^22^ are sometimes included in MENA definitions.

Based on the published literature,^24^, ^25^, ^26^, ^27^ we intended to evaluate whether the gray literature search and languages used in the literature search were dependent on the authors' origin, to the geographical coverage, and to the health topic of the SRs. Do authors from the region search additional grey literature sources and for languages relevant to the region? We also tested whether the impact factor of the journal where the SRs were published, and the year of publication could also influence the comprehensiveness of the literature search. The Statistical Package for the Social Sciences (SPSS) version 25 (SPSS, Chicago, IL, USA) was used to perform Fisher's exact test to assess the significance of the association between our variables. Association statistical significance threshold was at *P *< 0.05 (two‐tailed).

## 3 RESULTS

**FIGURE 1:**
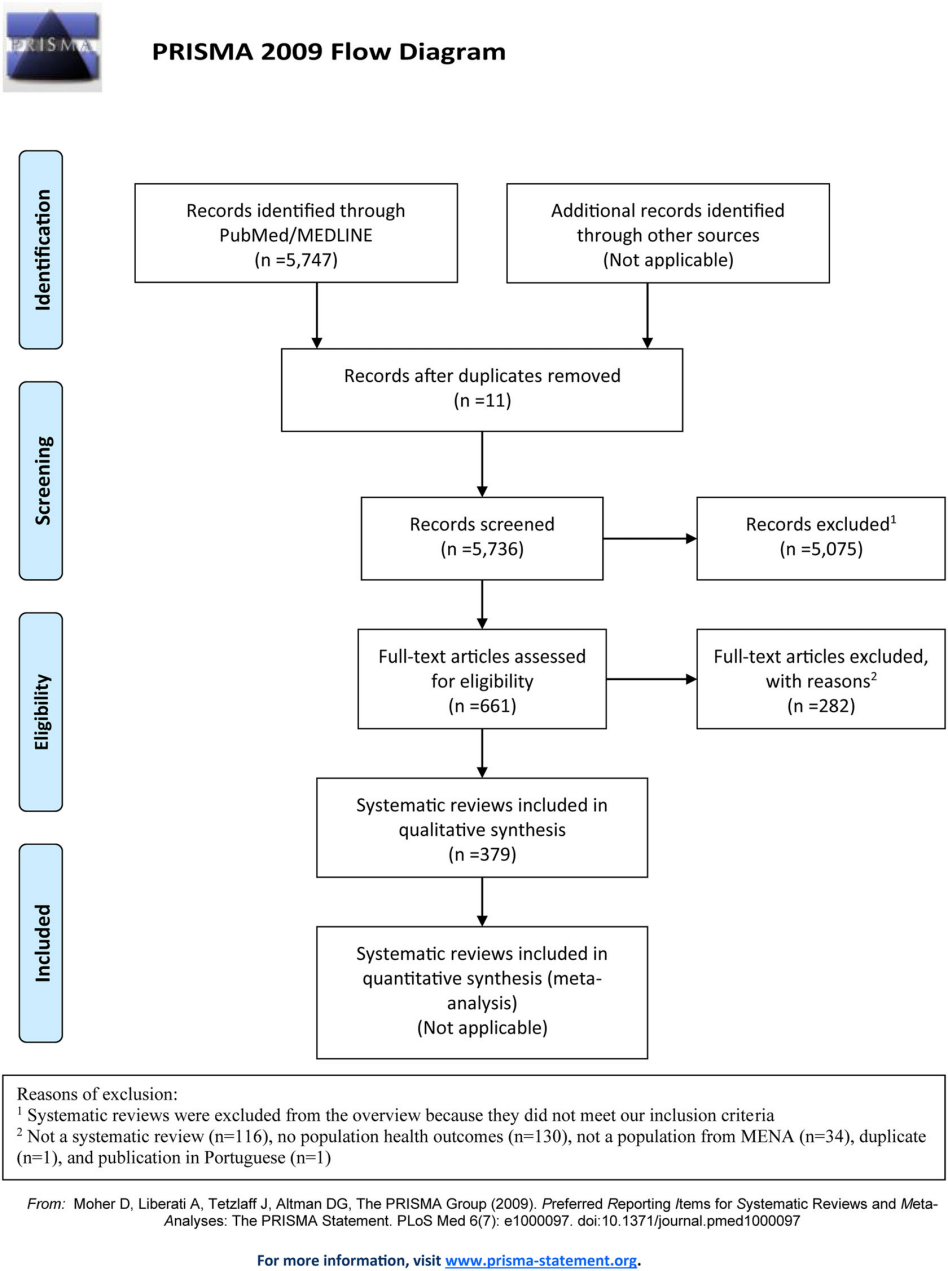
PRISMA 2009 flowchart

The initial literature search identified 5 747 articles. Thereafter, double multi‐stage screening included a cohort of 379 SRs (Figure 1).

Literature sources, which were reported in the SRs on population health in MENA were both commercial (Supplementary Material S3) and noncommercial. Commercial literature sources were a digital library, search engines, and publishers' and journals' websites. Almost all SRs searched the Medline database (97.4%, 369/379). Among them, 56 SRs reported searching PubMed, which includes Medline database, and Medline (sometimes reported through OVID). EMbase was searched by 138 SRs (36.4%), Web of Science Knowledge by 61 SRs (16.1%), and Scopus by 49 SRs (12.9%).

Gray literature sources were governmental (e.g. the website of the Ministry of Health, Population, and Hospital Reform Algeria), nongovernmental (e.g. Iraq Academic Scientific Journals digital library), and academic (e.g. Arabic Collections Online (ACO) – New York University). Google and Google Scholar were searched by 34 SRs (9.0%) and 85 SRs (22.4%), respectively.

Few SRs (10.3%, 39/379) reporting population health data on MENA countries conducted comprehensive literature searches allowing them to identify primary studies (gray and non‐gray literature) reported in languages other than English relevant to MENA. The comprehensiveness of the search did not statistically differ according to authors' affiliations, SRs' geographical coverage, and JIF.

**FIGURE 2:**
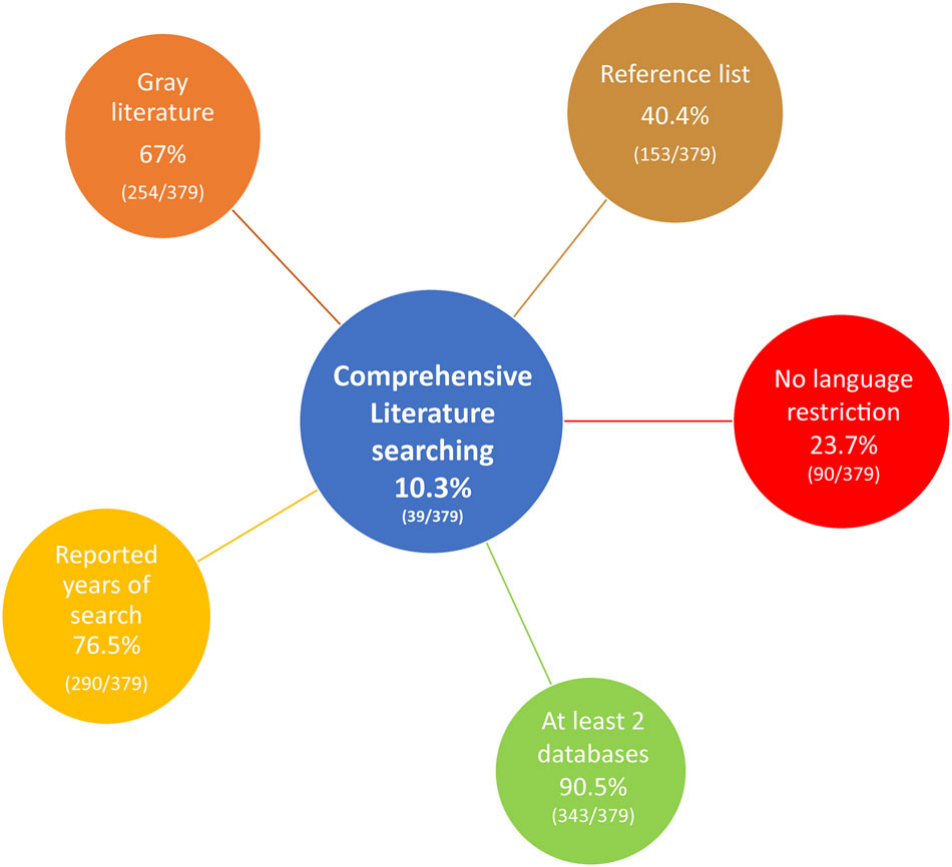
Literature search strategies utilized by systematic reviews in reporting population health data on the Middle East and North Africa

The vast majority of the SRs reporting data on population health in MENA countries searched at least two electronic databases (90.5%, 343/379), searched for gray literature (67.0%, 254/379), and reported the years of their literature search (76.5%, 290/379) (Figure 2) as recommended by AMSTAR.^5^ Multisource searching appears to be well integrated into the methodology of the published SRs. Nevertheless, one‐third of the SRs did not search for gray literature, suggesting room for methodological improvement in future reviews. For the 254 SRs included in our overview, which conducted gray literature searching, 19,823 primary studies were included by them which were relevant to SRs. Out of these 19,823 primary studies, there were 3042 studies (15.3%) which were reported as being identified from gray literature sources. This emphasizes the importance of gray literature searching. It appears that authors from the MENA region examined statistically additional gray literature sources than authors from the Western countries (Table 1). When authors from MENA and Western countries collaborate, a higher proportion searched for gray literature compared to those authors from Western countries who did not collaborate with MENA authors.

**TABLE 1:** Gray literature search and languages used in the included systematic reviews according to their characteristics

	Gray literature search		Languages used					
Stratified analysis	Yes (%, *n*)	*P* value*	No restriction (%, *n*)	English and 1 or 2 relevant languages (%, *n*)	English or English and not relevant languages (%, *n*)	Not mentioned (%, *n*)	*P* value*	Total (%, *n*)
Author affiliation								
Inside MENA	75.0% (84)	0.022	17.9% (20)	10.7% (12)	34.8% (39)	36.6% (41)	0.084	100% (112)
Collaboration	69.6% (80)		22.6% (26)	19.1% (22)	33.9% (39)	24.3% (28)		100% (115)
Outside MENA	59.2% (90)		28.9% (44)	15.1% (23)	33.6% (51)	22.4% (34)		100% (152)
Journal impact factor								
IF < 2	70.8% (121)	0.312	17.5% (30)	15.8% (27)	36.8% (63)	29.8% (51)	0.008	100% (171)
2 ≤ IF < 4	63.1% (82)		25.4% (33)	12.3% (16)	36.9% (48)	25.4% (33)		100% (130)
4 ≤ IF < 6	59.0% (23)		23.1% (9)	28.2% (11)	25.6% (10)	23.1% (9)		100% (39)
IF ≥ 6	71.8% (28)		46.2% (18)	7.7% (3)	20.5% (8)	25.6% (10)		100% (39)
Year of publication								
2008–2010	54.4% (31)	0.097	28.1% (16)	8.8% (5)	35.1% (20)	28.1% (16)	0.427	100% (57)
2011–2013	69.7% (83)		22.7% (27)	14.3% (17)	30.3% (36)	32.8% (39)		100% (119)
2014–2016	69.0% (150)		23.2% (47)	17.2% (35)	36.0% (73)	23.6% (48)		100% (203)
Geographical coverage								
North Africa	88.9% (8)	0.764	33.3% (3)	22.2% (2)	22.2% (2)	22.2% (2)	0.005	100% (9)
Eastern Africa	75.0% (3)		0.0% (0)	0.0% (0)	25.0% (1)	75.0% (3)		100% (4)
Country level	72.4% (63)		17.2% (15)	12.6% (11)	31.0% (27)	39.15% (34)		100% (87)
Middle East	70.4% (19)		22.2% (6)	14.8% (4)	33.3% (9)	29.6% (8)		100% (27)
MENA	69.2% (27)		25.6% (10)	17.9% (7)	23.1% (9)	33.3% (13)		100% (39)
Multiple regions	67.7% (21)		25.8% (8)	9.7% (3)	41.9% (13)	22.6% (7)		100% (31)
Arab countries	66.7% (22)		12.1% (4)	21.2% (7)	39.4% (13)	27.3% (9)		100% (33)
GCC	66.7% (8)		25.0% (3)	16.7% (2)	33.3% (4)	25.0% (3)		100% (12)
Africa	65.2% (15)		43.5% (10)	34.8% (8)	13.0% (3)	8.7% (2)		100% (23)
Global	62.8% (54)		30.2% (26)	15.1% (13)	33.7% (29)	20.9% (18)		100% (86)
Asia	50.0% (14)		17.9% (5)	0.0% (0)	67.9% (19)	14.3% (4)		100% (28)
Health topics								
Nutrition	85.7% (12)	0.011	7.1% (1)	0.0% (0)	57.1% (8)	35.7% (5)	0.376	100% (14)
Infectious disease	76.2% (99)		23.8% (31)	16.9% (22)	30.0% (39)	29.2% (38)		100% (130)
Oncology	75.0% (21)		17.9% (5)	14.3% (4)	39.3% (11)	28.6% (8)		100% (28)
Mental health	69.2% (18)		15.4% (4)	23.1% (6)	38.5% (10)	23.1% (6)		100% (26)
Other	59.0% (85)		25.0% (36)	13.2% (19)	35.4% (51)	26.4% (38)		100% (144)
Cardiovascular disease	52.9% (9)		17.6% (3)	17.6% (3)	35.3% (6)	29.4% (5)		100% (17)
Diabetes	50.0% (10)		50.0% (10)	15.0% (3)	20.0% (4)	15.0% (3)		100% (20)
Total	67.0% (254)		23.7% (90)	15.0% (57)	34.0% (129)	27.2% (103)		100% (379)

^*^Fisher's exact test was used to assess the significance of the association between our variables.

Gray literature search was not statistically associated with the languages used for the search. Not searching for gray literature does not necessarily mean that the search did not include words relevant to MENA. To the same effect, if search strategies were inclusive of gray literature, they may not necessarily have searched for literature published in languages pertinent to MENA. Only 23.7% (90/379) of the SRs did not have any language restrictions during their literature search, and an additional 15.0% searched in English and in one or two languages relevant to MENA. Of note, Arabic, English, French, and/or Urdu are the primary official language and/or medium of instruction in universities in MENA.^9^ A vast majority (61.2%, 232/379) of the SRs reporting data on MENA did not report searching for literature in Arabic, French, and/or Urdu in addition to their literature search in English. It is safe to assume that these SRs likely overlooked primary studies published in these languages. For instance, one of the SRs we included in our overview^16^ had no language restriction and reported data on Djibouti, Somalia, Sudan, and Yemen. Out of the 51 reports included in the SR, two were in the French language (3.9%). Interestingly, these two primary studies published in the French language were the only ones reporting data on Djibouti, which is a country where the French language is the medium of instruction. The proportion of SRs with different language restriction status statistically differed according to JIF categories. Almost half of the SRs published in journals with JIF ≥6 had no language restriction in their literature search strategy. The highest proportion of SRs (46.2%, 18/39) with no language restriction was observed among JIF ≥6. Nevertheless, as the substantial proportion of SRs did not report their language restriction (28.2%), the assessment of language use in the literature search might be biased. Language restriction was not reported in 39.1% (34/87) of country‐level SRs and in 75.0% (3/4) of Eastern African region SRs. These were significantly the highest proportions observed for the geographical coverages.

Additionally, less than half of the SRs searched the reference lists of the included studies (40%). Searching the reference lists of the included studies was statistically associated with searching for gray literature (*P *< 0.001): 93% of those not searching the reference lists of their included studies were also not searching for gray literature. In comparison, 75% of those seeking the reference lists of their included studies searched for gray literature. Searching reference lists was not statistically associated with author affiliation, SRs' geographical coverage, or JIF.

## 4 DISCUSSION

In this meta‐research study, we identify that a minority of SRs reporting population health data on MENA countries utilized a comprehensive search strategy combining searches of at least two databases and reference lists, a search for gray literature, and a search in languages relevant to MENA. More specifically, most SRs did search for at least two databases and gray literature. However, only a few searched the reference lists of included studies and searched in languages relevant to MENA.

Searching for literature (gray or nongray) predominantly in English may lead to missing data which may be published in other languages. This is of importance for research produced in MENA countries where English is not an official language and in many cases is not the medium of instruction in universities and colleges.^9^ For instance, searching only English sources when an SR reports epidemiological data on North African countries is profoundly incomplete because English is not a language commonly spoken in these countries and a substantial amount of literature is published in the French language.

In order to support improved literature searching pertaining to population health data in MENA, we have published the languages spoken and used in colleges and universities within the MENA countries.^9^ Additionally, with this manuscript, we also provided the lists of literature sources (including gray literature sources) used by SR authors reporting data on population health in MENA. The impact of searching only in the English language for epidemiological reviews covering the MENA region could be potential avenues for future investigation.

In addition, not searching the reference list of included primary studies may also lead to missing data. Researchers publishing primary studies conducted in MENA might be citing other studies from gray or nongray literature that are not indexed in the searched databases.

One study conducted in 2014 including SRs reported that 82% of epidemiological SRs searched at least two databases, 1% searched for gray literature, 78% searched the reference lists of included studies, and 39% reported no language restriction.^28^ In contrast, our study findings suggest that SRs' authors were reporting data on MENA search more frequently for gray literature (67%) to identify research not disseminated in journals controlled by commercial publishers. We observed that all SRs, however, did not extend their search to include languages relevant to MENA. Consequently, these published SRs may be missing essential data pertinent to the region.

We make the point that authors' adherence to reporting and methodological guidelines such as PRISMA and AMSTAR will improve the quality of the SRs reporting data on MENA. Emphasis must be made on the importance of a comprehensive literature search, particularly for this region since research is disseminated in languages other than English. We recommend that literature searches should be extended to Arabic, French, and/or Urdu, depending on the geographical coverage of the epidemiological SRs.

We believe this is the first study that evaluates the comprehensiveness of literature search strategies utilized in SRs reporting on population health data in MENA. We aimed to estimate the proportion of the included systematic reviews that comprehensively conducted their literature search, and to assess the differences between these systematic reviews according to their search. Consequently, we did not conduct an overview of systematic reviews in order to report a narrative synthesis and quantitative summaries about the epidemiology of health topics. Our study's strength is the large and representative sample of published SRs reporting population health data in MENA. The cohort of SRs on MENA population health was rigorously identified through a comprehensive search on PubMed, which is recognized as the most extensive public digital archive for biomedical journals.^29^ Including articles indexed on PubMed between 2008 and 2016 however, is not the same as including those published between 2008 and 2016.^28^ Consequently, we may have missed some SRs published in 2016 but indexed with a delay. Additionally, because our search strategy was built with MeSH and Title/Abstract terms, we may have missed some relevant SRs which did not include MENA country names in their title and abstract if these SRs were not indexed adequately in MEDLINE using the MeSH terms. Nevertheless, including fewer articles published would unlikely affect our overall findings and conclusions. Our assessment of the search strategy was based on the reporting quality of the SRs. Hence, we may have been conservative in our evaluation of the methodology of some SRs due to a lack of comprehensiveness in reporting. Furthermore, the assessment was conducted using the original version of the AMSTAR checklist.^6^ A new version of the AMSTAR checklist has been published;^5^ however, no significant differences between the two versions have been noticed for literature search strategy assessment of SRs including non‐randomized studies.

Though the vast majority of SRs reporting data on population health in MENA carried‐out multi‐source search, we identified weaknesses in their literature search strategies. The literature search was limited by saying quality, language restrictions during the search and lack of reference list search. We recommend that SRs adhere to the reporting guidelines^30^, ^31^, ^32^ and expand the search strategy to include languages relevant to MENA and to be inclusive of reference lists. This will help to improve the evidence‐based study on MENA population health problems, which is essential to develop health promotion and prevention strategies to tackle these issues through informed, evidence‐based decision making. We also recommend that editors of peer‐reviewed journals should require completed checklists supporting the submitted SRs^5^, ^6^, ^30^ to ensure the rigor of their methodology and reporting.

## Supporting information

Supporting Information

Supporting Information

Supporting Information
