# Supplementary material for: Strengthening literature search strategies for systematic reviews reporting population health in the Middle East and North Africa: A meta‐research study
Source: J Evid Based Med. 2020 May 24;13(3):192–8. doi: 10.1111/jebm.12394 (PMC7497175; doi:10.1111/jebm.12394)
Supplement: Supplementary file 1 — Supporting Information [file JEBM-13-192-s001.docx]

**Search strategy for PubMed**

("Qatar"[Mesh] OR "Bahrain"[Mesh] OR "Oman"[Mesh] OR "Saudi Arabia"[Mesh] OR "Kuwait"[Mesh] OR "United Arab Emirates"[Mesh] OR "Yemen"[Mesh] OR "Egypt"[Mesh] OR "Jordan"[Mesh] OR "Lebanon"[Mesh] OR "Syria"[Mesh] OR "Iraq"[Mesh] OR "Algeria"[Mesh] OR "Libya"[Mesh] OR "Morocco"[Mesh] OR "Tunisia"[Mesh] OR "Djibouti"[Mesh] OR "Sudan"[Mesh] OR "South Sudan"[Mesh] OR "Pakistan"[Mesh] OR "Africa, Northern"[Mesh] OR "Africa, Eastern"[Mesh] OR "middle east"[Mesh] OR "Arabs"[Mesh] OR "UAE"[Title/Abstract] OR "U.A.E"[Title/Abstract] OR "Emirat*"[Title/Abstract] OR "United Arab Emirates"[Title/Abstract] OR "Qatar*"[Title/Abstract] OR "Oman*"[Title/Abstract] OR "Saudi Arabia*"[Title/Abstract] OR "Saudi*"[Title/Abstract] OR "Kuwait*"[Title/Abstract] OR "Bahrain*"[Title/Abstract] OR "Yemen*"[Title/Abstract] OR "Egypt*"[Title/Abstract] OR "Jordan*"[Title/Abstract] OR "Leban*"[Title/Abstract] OR "Syria*"[Title/Abstract] OR "Iraq*"[Title/Abstract] OR "West Bank*"[Title/Abstract] OR "Gaza*"[Title/Abstract] OR "Palestin*"[Title/Abstract] OR "Algeria*"[Title/Abstract] OR "Libya*"[Title/Abstract] OR "Morocc*"[Title/Abstract] OR "Tunis*"[Title/Abstract] OR "Djibouti*"[Title/Abstract] OR "Sudan*"[Title/Abstract] OR "South Sudan*"[Title/Abstract] OR "Pakistan*"[Title/Abstract] OR "North Africa*"[Title/Abstract] OR "North-Africa*"[Title/Abstract] OR ("Africa"[Title/Abstract] AND "Northern"[Title/Abstract]) OR "Northern Africa"[Title/Abstract] OR "East Africa"[Title/Abstract] OR ("Africa"[Title/Abstract] AND "Eastern"[Title/Abstract]) OR "Maghreb"[Title/Abstract] OR "Maghrib"[Title/Abstract] OR "Arab*"[Title/Abstract] OR "Bedouin*"[Title/Abstract] OR "Gulf Cooperation Council"[Title/Abstract] OR "GCC"[Title/Abstract] OR "Middle East"[Title/Abstract])
