# Supplementary material for: Strengthening literature search strategies for systematic reviews reporting population health in the Middle East and North Africa: A meta‐research study
Source: J Evid Based Med. 2020 May 24;13(3):192–8. doi: 10.1111/jebm.12394 (PMC7497175; doi:10.1111/jebm.12394)
Supplement: Supplementary file 2 — Supporting Information [file JEBM-13-192-s002.docx]

## List of included systematic reviews reporting data on population health in the Middle East and North Africa, 2008-2016

| **Citation** | **Journal** | **Reported type of review** | **Literature Search Period** |
| --- | --- | --- | --- |
| AlMarri, 2009 (1) | Int J Psychol | Systematic Review | 1975-2007 |
| Popova, 2016 (2) | Trop Med Int Health | Systematic Review | 1983-2014 |
| Ghandour, 2016 (3) | Int J Drug Policy | Not Specified | 1993-2014 |
| Francis, 2014 (4) | Trop Med Int Health | Systematic Review & Meta-Analysis | Inception-2013 |
| John, 2013 (5) | Asian Pac J Cancer Prev | Not Specified | Inception-2012 |
| Degenhardt, 2011 (6) | Drug Alcohol Depend | Systematic Review | 1990-2008 |
| Degenhardt, 2014 (7) | Addiction | Not Specified | Unmentioned |
| Alsanosy, 2014 (8) | Asian Pac J Cancer Prev | Not Specified | Unmentioned |
| Harbour, 2011 (9) | East Mediterr Health J | Not Specified | Unmentioned |
| Almutairi, 2014 (10) | J Community Health | Not Specified | Inception-Current |
| Shah, 2015 (11) | Pak J Med Sci | Not Specified | Unmentioned |
| Abdullah, 2013 (12) | Int J Environ Res Public Health | Not Specified | 1987-2010 |
| Kassim, 2015 (13) | Biomed Res Int | Systematic Review | 1806-Current |
| Akl, 2011 (14) | BMC Public Health | Systematic Review | 1950-2008 |
| Akl, 2013 (15) | Harm Reduct J | Systematic Review | 1950-Unmentioned |
| Hassen, 2012 (16) | JBI Libr Syst Rev | Systematic Review | 1988-2011 |
| El-Zaemey, 2015 (17) | Int J Occup Environ Med | Systematic Review | Inception-2014 |
| Kakde, 2012 (18) | Public Health | Systematic Review | 1715-Unmentioned |
| Katelaris, 2012 (19) | Clin Exp Allergy | Not Specified | 1980-2010 |
| Aldrees, 2011 (20) | Saudi Dent J | Meta-Analysis | 1966-2008 |
| Sabbagh, 2012 (21) | Saudi Dent J | Systematic Review | 1980-2010 |
| Yammine, 2013 (22) | Clin Anat | Systematic Review & Meta-Analysis | Inception-2013 |
| Ameh, 2016 (23) | Lupus | Systematic Review | 1990-2015 |
| Lip, 2012 (24) | Chest | Systematic Review | 1990-2010 |
| Alshaikh, 2016 (25) | J Environ Public Health | Systematic Review | 2000-2015 |
| Deek, 2015 (26) | Collegian | Not Specified | Inception-Unmentioned |
| Benamer, 2009 (27) | J Neurol Sci | Systematic Review | 1950-2008 |
| Tran, 2010 (28) | J Neurol Sci | Not Specified | 1980-2007 |
| Behrouz, 2016 (29) | Eur J Neurol | Not Specified | 1994-2014 |
| Khan, 2013 (30) | J Thromb Thrombolysis | Not Specified | Inception-Current |
| Yamamoto, 2014 (31) | Int J Hyg Environ Health | Systematic Review | 1990-2012 |
| Nasser, 2015 (32) | Int J Occup Med Environ Health | Not Specified | 2000-2013 |
| Gonzalez, 2016 (33) | PLoS One | Systematic Review & Meta-Analysis | 1995-2015 |
| Akeroyd, 2015 (34) | World J Cardiol | Systematic Review | 1966-2015 |
| Donnelly, 2015 (35) | J Immigr Minor Health | Not Specified | Unmentioned |
| Barakat, 2012 (36) | Vasc Health Risk Manag | Not Specified | Unmentioned |
| Raza, 2013 (37) | Obes Facts | Systematic Review | 1971-2011 |
| Aljefree, 2015 (38) | Food Nutr Res | Systematic Review | 1990-2015 |
| Mehio Sibai, 2010 (39) | Ann Nutr Metab | Not Specified | Unmentioned |
| Nasreddine, 2014 (40) | J Med Liban | Not Specified | Unmentioned |
| Smythe, 2016 (41) | Trop Med Int Health | Systematic Review & Meta-Analysis | 1960-2015 |
| Vynnycky, 2016 (42) | PLoS One | Systematic Review | 1990-2011 |
| Deckers, 2012 (43) | PLoS One | Systematic Review | 1990-2010 |
| Jayawardena, 2012 (44) | BMC Public Health | Systematic Review & Meta-Analysis | 1980-2011 |
| Bos, 2013 (45) | BMC Public Health | Systematic Review | 1990-2012 |
| Zabetian, 2013 (46) | Diabetes Res Clin Pract | Not Specified | 1990-2012 |
| Lamri, 2014 (47) | Global Health | Not Specified | Inception-Current |
| NCD Risk Factor Collaboration, 2016 (48) | Lancet | Not Specified | 1950-2013 |
| Mbanya, 2015 (49) | Prim Care Diabetes | Systematic Review | 1999-2014 |
| Meo, 2016 (50) | J Pak Med Assoc | Systematic Review | Inception-Current |
| Zayed, 2016 (51) | Int J Clin Pract | Systematic Review | Inception-2015 |
| Usher-Smith, 2012 (52) | Diabetologia | Systematic Review | Inception-2011 |
| Danaei, 2011 (53) | Lancet | Systematic Review | 1980-2008 |
| Alhyas, 2012 (54) | PLoS One | Systematic Review | 1947-2010 |
| Meo, 2016 (55) | J Pak Med Assoc | Not Specified | Unmentioned |
| Alkabab, 2015 (56) | Int J Infect Dis | Not Specified | Unmentioned |
| Hassan, 2014 (57) | Eur Rev Med Pharmacol Sci | Not Specified | Unmentioned |
| Yako, 2016 (58) | Diabetes Res Clin Pract | Systematic Review & Meta-Analysis | Inception-2014 |
| Hamzeh, 2016 (59) | Hla | Meta-Analysis | Inception-2015 |
| Hamzeh, 2015 (60) | Tissue Antigens | Meta-Analysis | Unmentioned |
| Alharbi, 2014 (61) | Diabetes Res Clin Pract | Systematic Review & Meta-Analysis | 1979-2011 |
| Al-Khudairy, 2013 (62) | Nutrients | Systematic Review | Inception-2013 |
| Fleming, 2009 (63) | J Transcult Nurs | Not Specified | 1951-Current |
| Motlagh, 2009 (64) | Eur J Cardiovasc Prev Rehabil | Systematic Review | 1980-2005 |
| Khader, 2015 (65) | Rev Environ Health | Systematic Review | 2000-2014 |
| DeNicola, 2015 (66) | Ann Glob Health | Not Specified | 2008-Current |
| Singh, 2016 (67) | J Gastroenterol Hepatol | Systematic Review & Meta-Analysis | 1991-2014 |
| Gautret, 2015(68) | Travel Med Infect Dis | Not Specified | 1980-2014 |
| Olusanya, 2015 (69) | PLoS One | Systematic Review & Meta-Analysis | 1990-2014 |
| Moldecky, 2012 (70) | Gastroenterology | Systematic Review | 1950-2010 |
| Devanarayana, 2015(71) | J Pediatr Gastroenterol Nutr | Not Specified | 1948-2014 |
| Sperber, 2016 (72) | Gut | Not Specified | Unmentioned |
| Ashtari, 2015 (73) | World J Hepatol | Not Specified | Unmentioned |
| Jiang, 2013 (74) | PLoS One | Not Specified | 2002-2012 |
| de Menthon, 2009 (75) | Arthritis Rheum | Systematic Review & Meta-Analysis | 1973-2007 |
| Romdhane, 2012 (76) | Orphanet J Rare Dis | Systematic Review | Inception-2012 |
| Doss, 2016 (77) | Sci Rep | Not Specified | Inception-2016 |
| Romdhane, 2011 (78) | Am J Med Genet A | Not Specified | Inception-2010 |
| Sabahelzain, 2014 (79) | Pan Afr Med J | Not Specified | Unmentioned |
| Habeb, 2013 (80) | Libyan J Med | Systematic Review | Inception-2013 |
| Mahdieh, 2016 (81) | Blood Rev | Not Specified | Unmentioned |
| Bader, 2016 (82) | PLoS One | Systematic Review | Unmentioned |
| Gaikwad, 2014 (83) | Thromb Res | Not Specified | Unmentioned |
| Inhorn, 2015 (84) | Hum Reprod Update | Not Specified | 2000-2014 |
| Mandil, 2013 (85) | Int J Epidemiol | Not Specified | 1996-2012 |
| Tajvar, 2013 (86) | Australas J Ageing | Systematic Review | Inception-Current |
| Benova, 2014 (87) | PLoS One | Not Specified | 1992-2013 |
| Salmon-Rosseau, 2016 (88) | Med Mal Infect | Not Specified | Inception-2015 |
| Tosson, 2011 (89) | J Matern Fetal Neonatal Med | Not Specified | 1990-2009 |
| Gautret, 2016 (90) | Int J Infect Dis | Not Specified | 1980-2016 |
| Prasad, 2015 (91) | PLoS One | Systematic Review | 1980-2013 |
| DeAntonia, 2016(92) | Int J Pediatr Otorhinolaryngol | Systematic Review | 1992-2011 |
| Musallam, 2016 (93) | Epidemiol Infect | Systematic Review | Inception-Current |
| Naseer, 2014 (94) | J Coll Physicians Surg Pak | Not Specified | 1995-2010 |
| Buckle, 2012 (95) | J Glob Health | Systematic Review | 1980-2009 |
| Azamatullah, 2015 (96) | J Glob Health | Systematic Review | 1964-2013 |
| Coffin, 2010 (97) | Int J Drug Policy | Not Specified | 1995-2007 |
| Kenyon, 2013 (98) | Am J Obstet Gynecol | Systematic Review | Inception-Current |
| Manezhe, 2015 (99) | J Antimicrob Chemother | Systematic Review | Unmentioned |
| Vanderburg, 2014 (100) | PLoS Negl Trop Dis | Systematic Review | Unmentioned |
| Steer, 2009 (101) | Lancet Infect Dis | Systematic Review | 1990-2009 |
| Khedmat, 2013 (102) | Caspian J Intern Med | Not Specified | Unmentioned |
| Eshragian, 2014 (103) | World J Gastroenterol | Systematic Review | Unmentioned |
| Dean, 2012 (104) | PLoS Negl Trop Dis | Systematic Review | 1990-2010 |
| Shibi, 2009 (105) | Int J Antimicrob Agents | Not Specified | 1990-2007 |
| Hung, 2013 (106) | Int J Infect Dis | Not Specified | 1995-2011 |
| Fares, 2011 (107) | J Glob Infect Dis | Not Specified | Unmentioned |
| Horton, 2016 (108) | PLoS Med | Systematic Review & Meta-Analysis | 1993-2016 |
| Ghenghesh, 2015 (109) | Comp Immunol Microbiol Infect Dis | Not Specified | 1995-2014 |
| Tansarli, 2014 (110) | J Antimicrob Chemother | Systematic Review | 2005-2013 |
| Areeshi, 2014 (111) | J Infect Dev Ctries | Not Specified | 2002-2013 |
| Sangare, 2015 (112) | Med Mal Infect | Not Specified | 1996-2014 |
| Abdulgader, 2015 (113) | Front Microbiol | Systematic Review | Inception-2014 |
| Mamishi, 2014 (114) | Iran J Microbiol | Systematic Review | 1993-2013 |
| Jaiswal, 2014 (115) | PLoS One | Systematic Review | 1970-2014 |
| Gu, 2015 (116) | Epidemiol Infect | Not Specified | 1998-2012 |
| Kahsay, 2016 (117) | BMC Res Notes | Not Specified | 2001-2014 |
| Berrazeg, 2014 (118) | Euro Surveill | Meta-Analysis | 2009-2012 |
| Velayati, 2015 (119) | Int J Mycobacteriol | Not Specified | 1984-2014 |
| Al-Tawfiq, 2015 (120) | J Chemother | Systematic Review | Unmentioned |
| Chakrabarti, 2011 (121) | Med Mycol | Not Specified | 1970-2010 |
| van de Sande, 2013 (122) | PLoS Negl Trop Dis | Systematic Review & Meta-Analysis | Inception-Current |
| Romani, 2015 (123) | Lancet Infect Dis | Systematic Review | 1985-2014 |
| Araj, 2014 (124) | J Med Liban | Not Specified | Unmentioned |
| D'Acremont, 2010 (125) | Malar J | Systematic Review | 1989-2009 |
| Rahimi, 2014 (126) | Malar J | Systematic Review & Meta-Analysis | 1900-Current |
| Coleman, 2014 (127) | PLoS One | Not Specified | 1980-2012 |
| Pappas, 2009 (128) | Int J Parasitol | Not Specified | 1999-2008 |
| Alsammani, 2016 (129) | J Parasit Dis | Not Specified | Unmentioned |
| Al-Salem, 2016 (130) | Parasit Vectors | Not Specified | 1955-2016 |
| Youssef, 2008 (131) | J Egypt Soc Parasitol | Not Specified | Unmentioned |
| Roberts, 2011 (132) | J Glob Health | Not Specified | 1950-2010 |
| Chanda, 2013 (133) | Malar J | Systematic Review | Unmentioned |
| Ansarie, 2014 (134) | J Pak Med Assoc | Not Specified | 2003-2013 |
| DeAntonio, 2016 (135) | Hum Vaccin Immunother | Systematic Review | Unmentioned |
| Alqahtani, 2015 (136) | Clin Microbiol Infect | Not Specified | Inception-2014 |
| Gasim, 2015 (137) | Virol J | Not Specified | 1980-2014 |
| Van-Lume, 2013 (138) | Rev Saude Publica | Systematic Review | 1990-2011 |
| Bosan, 2010 (139) | Not Specified | Not Specified | Unmentioned |
| Azevedo, 2016 (140) | J Int AIDS Soc | Systematic Review & Meta-Analysis | Unmentioned |
| Schweiter, 2015 (141) | Lancet | Systematic Review | 1965-2013 |
| Ali, 2011(142) | Virol J | Systematic Review | Unmentioned |
| Ott, 2012 (143) | BMC Infect Dis | Not Specified | 1980-2007 |
| Ezzikouri, 2013 (144) | Liver Int | Not Specified | Inception-2012 |
| Gasim, 2013 (145) | Arab J Gastroenterol | Not Specified | Unmentioned |
| Babanejad, 2016 (146) | Hepat Mon | Systematic Review & Meta-Analysis | 2000-2015 |
| Bajubair, 2008 (147) | Saudi Med J | Not Specified | 2000-2005 |
| Ali, 2009 (148) | Int J Infect Dis | Not Specified | 1994-2007 |
| Lehman, 2009 (149) | Int J Cancer | Systematic Review & Meta-Analysis | 1980-2007 |
| Nelson, 2011 (150) | Lancet | Systematic Review | Inception-2011 |
| Gasim, 2013 (151) | J Infect Dev Ctries | Not Specified | Unmentioned |
| Bashour, 2016 (152) | East Mediterr Health J | Not Specified | Unmentioned |
| Riaz, 2011 (153) | Virol J | Not Specified | 1983-2010 |
| Raja, 2008 (154) | J Microbiol Immunol Infect | Not Specified | 1970-2005 |
| Stern, 2008 (155) | BMC Med Res Methodol | Not Specified | 1989-2006 |
| Umar, 2009 (156) | J Coll Physicians Surg Pak | Not Specified | 1992-2008 |
| Waheed, 2009 (157) | World J Gastroenterol | Systematic Review | 1994-2009 |
| Attaullah, 2011 (158) | Virol J | Systematic Review | 1996-2011 |
| Sievert, 2011 (159) | Liver Int | Systematic Review | Unmentioned |
| Alavian, 2012 (160) | Hepat Mon | Meta-Analysis | Inception-2011 |
| Ramia, 2012 (161) | Infection | Not Specified | 2000-2010 |
| Karoney, 2013 (162) | Pan Afr Med J | Not Specified | 1995-Unmentioned |
| Abozaid, 2013 (163) | Ann Saudi Med | Systematic Review | Inception-2011 |
| Ezzikouri, 2013 (164) | J Med Virol | Not Specified | Inception-2012 |
| Mohd Hanafiah, 2013 (165) | Hepatology | Systematic Review | 1980-2007 |
| Mohamoud, 2013 (166) | BMC Infect Dis | Systematic Review | Inception-Unmentioned |
| Reker, 2014 (167) | Int J Infect Dis | Not Specified | 2008-2012 |
| Bruggmann, 2014 (168) | J Viral Hepat | Not Specified | 1990-2013 |
| Gower, 2014 (169) | J Hepatol | Not Specified | 2000-Unmentioned |
| El-Ghitany, 2015 (170) | Liver Int | Meta-Analysis | 1989-2013 |
| Chemaitelly, 2015 (171) | PLoS One | Systematic Review & Meta-Analysis | 1989-2015 |
| Fadlalla, 2015 (172) | PLoS One | Systematic Review & Meta-Analysis | 1980-Unmentionned |
| Riou, 2016 (173) | J Viral Hepat | Systematic Review & Meta-Analysis | 2000-2014 |
| Ghaderi-Zefrehi, 2016 (174) | Hepat Mon | Systematic Review & Meta-Analysis | 1995-2016 |
| Sadeghi, 2016 (175) | Hepat Mon | Systematic Review & Meta-Analysis | 2000-2015 |
| Chaabna, 2016 (176) | PLoS One | Systematic Review & Meta-Analysis | Inception-2015 |
| Mohamoud, 2016 (177) | Int J Infect Dis | Systematic Review & Meta-Analysis | Unmentioned |
| Amini, 2013 (178) | Hepat Mon | Systematic Review & Meta-Analysis | Inception-2010 |
| Alavian, 2016 (179) | Hepat Mon | Systematic Review | 1989-2015 |
| de Martel, 2015 (180) | Hepatology | Systematic Review | 1989-2014 |
| Jacobson, 2010 (181) | Vaccine | Not Specified | 1990-2005 |
| Itani, 2014 (182) | Vaccine | Not Specified | Unmentioned |
| Yazbek, 2016 (183) | Infection | Not Specified | 2000-2014 |
| Parashar, 2009 (184) | J Infect Dis | Not Specified | 1990-2004 |
| Khoury, 2011 (185) | BMC Infect Dis | Not Specified | 1999-2009 |
| Miles, 2012 (186) | Vaccine | Systematic Review | 1983-2009 |
| Kawai, 2012 (187) | Vaccine | Systematic Review | 2000-2011 |
| Tate, 2012 (188) | Lancet Infect Dis | Systematic Review & Meta-Analysis | 2001-2011 |
| Jroundi, 2015 (189) | Arch Public Health | Not Specified | 1997-2014 |
| Abubaker, 2016 (190) | East Mediterr Health J | Not Specified | 1995-2015 |
| Mathur, 2014 (191) | PLoS One | Not Specified | 1997-2013 |
| Lai, 2016 (192) | Lancet Infect Dis | Systematic Review | 1997-2015 |
| Tricco, 2013 (193) | Influenza Other Respir Viruses | Systematic Review | 2009-2011 |
| Alsolamy, 2015 (194) | Crit Care Med | Not Specified | Unmentioned |
| Gautret, 2016 (195) | Travel Med Infect Dis | Systematic Review | 1980-2015 |
| Mathers, 2008 (196) | Lancet | Systematic Review | Inception-Current |
| Mathers, 2010 (197) | Lancet | Systematic Review | 2004-Unmentioned |
| Abu-Raddad, 2010 (198) | Aids | Not Specified | Inception-Current |
| Mumtaz, 2010 (199) | PLoS Med | Systematic Review | Unmentioned |
| Mumtaz, 2011 (200) | Sex Transm Infect | Not Specified | Inception-Current |
| Kouyoumjian, 2013 (201) | Int J STD AIDS | Systematic Review | Inception-Current |
| Mumtaz, 2013 (202) | Sex Transm Infect | Not Specified | Unmentioned |
| Singh, 2014 (203) | Int J Drug Policy | Systematic Review | Inception-2011 |
| Mumtaz, 2014 (204) | PLoS Med | Systematic Review | Inception-2013 |
| Poteat, 2016 (205) | J Acquir Immune Defic Syndr | Not Specified | 2012-2015 |
| Moradi, 2016 (206) | Iran J Psychiatry Behav Sci | Not Specified | Inception-Current |
| Lihana, 2012 (207) | AIDS Rev | Not Specified | 2000-2011 |
| Abu-Raddad, 2010 (208) | Epidemics | Not Specified | Inception-Current |
| Caceres, 2008 (209) | Sex Transm Infect | Not Specified | 2003-2007 |
| Serbessa, 2016 (210) | Afr J AIDS Res | Not Specified | 2001-2004 |
| Bruni, 2010 (211) | J Infect Dis | Meta-Analysis | 1995-2009 |
| Smith, 2008 (212) | J Adolesc Health | Not Specified | 1989-2014 |
| Ogembo, 2015 (213) | PLoS One | Systematic Review & Meta-Analysis | Inception-2013 |
| Seoud, 2012 (214) | J Low Genit Tract Dis | Not Specified | Unmentioned |
| Alkaiyat, 2013 (215) | Int J Public Health | Not Specified | Unmentioned |
| Furuya-k  Kanamori, 2016 (216) | BMC Infect Dis | Not Specified | Inception-2015 |
| Alhaeli, 2016 (217) | J Infect Public Health | Systematic Review | 2006-2014 |
| Humphrey, 2016 (218) | PLoS Negl Trop Dis | Systematic Review | Inception-2015 |
| Razavi, 2016 (219) | Int J Prev Med | Not Specified | 2010-2016 |
| Ghenghesh, 2013 (220) | Libyan J Med | Not Specified | 1970-2011 |
| Tanios, 2009 (221) | J Anxiety Disord | Not Specified | Unmentioned |
| Elzubeir, 2010 (222) | Educ Health (Abingdon) | Not Specified | 1998-2008 |
| Farah, 2009 (223) | J Atten Disord | Not Specified | Unmentioned |
| Alhraiwil, 2015 (224) | Neurosciences (Riyadh) | Systematic Review | Unmentioned |
| Alkhateeb, 2016 (225) | J Atten Disord | Systematic Review | Inception-Current |
| Bakare, 2011 (226) | Afr J Psychiatry (Johannesbg) | Not Specified | 2000-2009 |
| Salhia, 2014 (227) | Neurosciences (Riyadh) | Not Specified | Inception-2013 |
| Esan, 2016 (228) | Soc Psychiatry Psychiatr Epidemiol | Systematic Review | Inception-Current |
| Zahidie, 2013 (229) | J Coll Physicians Surg Pak | Not Specified | 2001-2011 |
| Travers, 2013 (230) | Expert Rev Pharmacoecon Outcomes Res | Systematic Review | 2001-2011 |
| Dardas, 2016 (231) | Issues Ment Health Nurs | Systematic Review | Inception-Current |
| Klainin, 2009 (232) | Int J Nurs Stud | Not Specified | 1998-2008 |
| Cheng, 2014 (233) | Cyberpsychol Behav Soc Netw | Meta-Analysis | 1950-Current |
| Nasser, 2011 (234) | Int J Psychiatry Clin Pract | Not Specified | Inception-2009 |
| Dimitry, 2012 (235) | Child Care Health Dev | Systematic Review | Unmentioned |
| Kronfol, 2014 (236) | Asian J Psychiatr | Not Specified | Unmentioned |
| Marie, 2016 (237) | Int J Ment Health Syst | Not Specified | Unmentioned |
| Hassan, 2016 (238) | Epidemiol Psychiatr Sci | Not Specified | Inception-2015 |
| Ayer, 2015 (239) | Trauma Violence Abuse | Systematic Review | 1990-2014 |
| Wells, 2016 (240) | Br J Psychiatry | Systematic Review | 2011-2015 |
| Rezaeian, 2010 (241) | Crisis | Not Specified | 1968-2008 |
| Morovatdar, 2013 (242) | Arch Suicide Res | Systematic Review & Meta-Analysis | Inception-2011 |
| Jordans, 2014 (243) | BMC Psychiatry | Not Specified | 2002-Current |
| Mars, 2014 (244) | BMC Public Health | Not Specified | 1998-2013 |
| Fuhr, 2014 (245) | Lancet Psychiatry | Systematic Review & Meta-Analysis | 1994-2013 |
| Shahid, 2008 (246) | Int J Inj Contr Saf Promot | #NULL! | Unmentioned |
| Al-Khateeb, 2014 (247) | Epilepsy Behav | Not Specified | 1990-2013 |
| Amawi, 2014 (248) | J Nerv Ment Dis | Not Specified | 1995-2012 |
| van de Meer, 2011 (249) | Osteoporos Int | Not Specified | 1990-Current |
| Badawi, 2012 (250) | J Public Health Res | Systematic Review | 1980-2012 |
| Bassil, 2013 (251) | Dermatoendocrinol | Systematic Review | 2000-2012 |
| Hilger, 2014 (252) | Br J Nutr | Systematic Review | 1990-2011 |
| Palacios, 2014 (253) | J Steroid Biochem Mol Biol | Systematic Review | 2003-2013 |
| Farrokhyar, 2015 (254) | Sports Med | Systematic Review & Meta-Analysis | Inception-2013 |
| Al-Daghri, 2016 (255) | J Steroid Biochem Mol Biol | Not Specified | 2011-2016 |
| Agarwal, 2009 (256) | World J Surg | Not Specified | Unmentioned |
| Mehboob, 2016 (257) | J Pak Med Assoc | Systematic Review | 2005-2015 |
| Hammad, 2016 (258) | J Transcult Nurs | Not Specified | 2003-2016 |
| Danaei, 2011 (259) | Lancet | Systematic Review | 1980-2008 |
| Hasan, 2014 (260) | Curr Hypertens Rev | Systematic Review | 1996-2013 |
| Neupane, 2014 (261) | Medicine (Baltimore) | Systematic Review & Meta-Analysis | 2000-2013 |
| Tailakh, 2014 (262) | Nurs Health Sci | Systematic Review | 1980-2011 |
| Nansseu, 2016 (263) | BMJ Open | Systematic Review & Meta-Analysis | Inception-2016 |
| Mabry, 2010 (264) | Diabet Med | Systematic Review | Unmentioned |
| Mirmiran, 2010 (265) | East Mediterr Health J | Not Specified | 1990-2007 |
| Karageorgi, 2013 (266) | J Obes | Not Specified | Inception-Current |
| Toselli, 2014 (267) | Eur J Public Health | Not Specified | 1984-2013 |
| Mistry, 2015 (268) | Public Health | Systematic Review | 1990-2013 |
| Alhyas, 2011 (269) | JRSM Short Rep | Systematic Review | 1947-2010 |
| Farzadfar, 2011 (270) | Lancet | Systematic Review | 1980-2008 |
| Bamimore, 2015 (271) | J Clin Lipidol | Not Specified | Unmentioned |
| Abou Abbas, 2015 (272) | Clin Obes | Systematic Review & Meta-Analysis | Inception-2014 |
| Sadat-Ali, 2012 (273) | Ann Saudi Med | Not Specified | 1952-2011 |
| Baddoura, 2011 (274) | J Clin Densitom | Not Specified | 1966-2009 |
| Najjar, 2010 (275) | Int J Surg | Not Specified | Unmentioned |
| Bhikoo, 2011 (276) | Cancers (Basel) | Systematic Review | Inception-2009 |
| Alhurishi, 2011 (277) | Asian Pac J Cancer Prev | Systematic Review | 1970-2011 |
| Corbex, 2014 (278) | Eur J Cancer | Not Specified | Unmentioned |
| Dubey, 2015 (279) | Asian Pac J Cancer Prev | Systematic Review | Unmentioned |
| Ting, 2010 (280) | Int J Gynecol Cancer | Not Specified | 1990-2007 |
| Alhazzari, 2016 (281) | Asian Pac J Cancer Prev | Systematic Review | Unmentioned |
| Ashtari, 2015 (282) | World J Hepatol | Not Specified | Unmentioned |
| Dubey, 2016 (283) | Chin J Cancer | Systematic Review | Unmentioned |
| Ahmed, 2013 (284) | J Oral Maxillofac Res | Not Specified | Unmentioned-2012 |
| Krishna Rao, 2013 (285) | Asian Pac J Cancer Prev | Not Specified | 2000-2012 |
| BenNasir, 2015 (286) | Saudi Dent J | Not Specified | 1946-2013 |
| Al-Jaber, 2016 (287) | Saudi Med J | Not Specified | 1985-Unmentionned |
| Sung, 2014 (288) | Gynecol Oncol | Systematic Review | 1990-2012 |
| Cherbal, 2012 (289) | Breast Dis | Not Specified | Unmentioned |
| Laraqui, 2015 (290) | Dis Markers | Not Specified | Unmentioned |
| Oluwgbemiga, 2012 (291) | Springerplus | Systematic Review | Unmentioned |
| Eng, 2014 (292) | PLoS Med | Systematic Review & Meta-Analysis | 1980-2014 |
| Midha, 2015 (293) | Am J Cancer Res | Systematic Review | 2004-Unmentioned |
| Irshad, 2015 (294) | Asian Pac J Cancer Prev | Meta-Analysis | Inception-Current |
| Shaik, 2015 (295) | Saudi J Gastroenterol | Not Specified | Inception-2014 |
| Huang, 2015 (296) | J Epidemiol | Not Specified | 1997-Unmentioned |
| Hussein, 2016 (297) | Infect Agent Cancer | Not Specified | Inception-Unmentionned |
| Baandrup, 2014 (298) | Eur J Cancer | Systematic Review & Meta-Analysis | Inception-2013 |
| Haddou Rahou, 2016 (299) | Health Qual Life Outcomes | Not Specified | Inception-2015 |
| Donnelly, 2013 (300) | Asian Pac J Cancer Prev | Not Specified | 2000-2011 |
| Alananzeh, 2016 (301) | Asia Pac J Oncol Nurs | Not Specified | Inception-Unmentioned |
| Peleteiro, 2008 (302) | Gac Sanit | Systematic Review | 1996-2006 |
| Almaguer, 2014 (303) | MEDICC Rev | Not Specified | Unmentioned |
| Hassanien, 2012 (304) | JRSM Short Rep | Systematic Review | 1950-2010 |
| Goleg, 2014 (305) | Int Urol Nephrol | Not Specified | 2003-2012 |
| Okpechi, 2016 (306) | PLoS One | Systematic Review & Meta-Analysis | 1980-2014 |
| McGrogan, 2011 (307) | Nephrol Dial Transplant | Systematic Review | 1980-2010 |
| Faust, 2009 (308) | J Urol | Meta-Analysis | 1980-2006 |
| Gwer, 2013 (309) | Paediatr Int Child Health | Not Specified | Unmentioned |
| Marin, 2016 (310) | Int J Epidemiol | Meta-Analysis | Inception-2015 |
| Benamer, 2009 (311) | Epilepsia | Systematic Review | 1950-2008 |
| Angalakuditi, 2011 (312) | Neuropsychiatr Dis Treat | Systematic Review | 1999-2010 |
| Bhalla, 2016 (313) | Seizure | Not Specified | Inception-Current |
| Benamer, 2014 (314) | J Neurol Sci | Systematic Review | 1946-Current |
| Webb, 2015 (315) | J Neurol Neurosurg Psychiatry | Systematic Review & Meta-Analysis | Inception-2014 |
| Benamer, 2010 (316) | J Headache Pain | Not Specified | 1950-2009 |
| Al-Hashel, 2008 (317) | Neuroepidemiology | Not Specified | Inception-2007 |
| Heydarpour, 2015 (318) | Neuroepidemiology | Systematic Review & Meta-Analysis | 1985-Unmentioned |
| Eskandarieh, 2016 (319) | Neuroepidemiology | Systematic Review | 1950-2014 |
| Benamer, 2009 (320) | J Neurol Sci | Systematic Review | 1950-2008 |
| Benamer, 2008 (321) | Mov Disord | Systematic Review | 1950-2007 |
| Benamer, 2015 (322) | Muscle Nerve | Systematic Review | Inception-2014 |
| Correia Guedes, 2010 (323) | Parkinsonism Relat Disord | Systematic Review | Inception-2008 |
| Amara, 2014 (324) | Global Health | Not Specified | 1980-2012 |
| Al-Qasem, 2011 (325) | East Mediterr Health J | Not Specified | Unmentioned |
| Ng, 2011 (326) | Obes Rev | Not Specified | 1990-2009 |
| Boutayeb, 2013 (327) | Int J Equity Health | Not Specified | Unmentioned |
| John, 2015 (328) | Oman Med J | Not Specified | Unmentioned |
| Nazeri, 2015 (329) | Thyroid | Systematic Review | 1964-2013 |
| Mirmiran, 2012 (330) | Iran J Public Health | Systematic Review | 1985-2011 |
| Nielsen, 2011 (331) | Int J Epidemiol | Meta-Analysis | 2004-2008 |
| Tsigga, 2012 (332) | J Epidemiol Glob Health | Not Specified | Unclear |
| Akhtar, 2016 (333) | Crit Rev Food Sci Nutr | Not Specified | Unmentioned |
| Musaiger, 2011 (334) | Int J Environ Res Public Health | Not Specified | 1990-2011 |
| Hirani, 2012 (335) | J Ayub Med Coll Abbottabad | Not Specified | 1991-2011 |
| Best, 2010 (336) | Food Nutr Bull | Not Specified | 2002-2009 |
| Stark, 2016 (337) | Prog Lipid Res | Systematic Review | Inception-2014 |
| Creo, 2015 (338) | Paediatr Int Child Health | Not Specified | 2006-2015 |
| Stoffaneller, 2015 (339) | Nutrients | Not Specified | 1990-2014 |
| Powles, 2013 (340) | BMJ Open | Systematic Review | 1980-Current |
| Sharma, 2016 (341) | Int Breastfeed J | Systematic Review | 1990-Unmentioned |
| Ahmad, 2016 (342) | J Pak Med Assoc | Systematic Review | 1945-2012 |
| Stevens, 2013 (343) | Ophthalmology | Not Specified | 1990-2010 |
| Bourne, 2013 (344) | Ophthalmic Epidemiol | Systematic Review | 1980-2012 |
| Cheng, 2014 (345) | PLoS One | Systematic Review & Meta-Analysis | Inception-2014 |
| Khairallah, 2014 (346) | Br J Ophthalmol | Not Specified | 1980-2012 |
| Gilbert, 2016 (347) | Ophthalmology | Systematic Review | 2000-Current |
| Khan, 2013 (348) | Saudi Med J | Meta-Analysis | 1999-2008 |
| Al-Bluwi, 2014 (349) | Int Dent J | Not Specified | Inception-2013 |
| Khan, 2014 (350) | Int Dent J | Systematic Review & Meta-Analysis | 1999-2012 |
| Al Agili, 2013 (351) | Saudi Dent J | Systematic Review | Unmentioned |
| Barzangi, 2014 (352) | Acta Odontol Scand | Not Specified | Inception-2012 |
| Al-Nasser, 2014 (353) | J Oral Pathol Med | Not Specified | Inception-2012 |
| Al-Harthi, 2013 (354) | Int Dent J | Not Specified | Inception-2011 |
| Mohamed Zaki, 2015 (355) | Pain Manag Nurs | Systematic Review | Inception-Current |
| Sisson, 2008 (356) | Obes Rev | Not Specified | 1996-2008 |
| Mabry, 2010 (357) | Obes Rev | Not Specified | Unmentioned |
| Ranasinghe, 2013 (358) | Int J Behav Nutr Phys Act | Systematic Review | Inception-2012 |
| Yammine, 2016 (359) | Perspect Public Health | Meta-Analysis | Inception-2015 |
| Mabry, 2016 (360) | BMC Public Health | Systematic Review | Inception-2016 |
| Loney, 2013 (361) | Glob Health Action | Not Specified | 1950-2012 |
| Adeloye, 2015 (362) | J Glob Health | Systematic Review & Meta-Analysis | 1990-2014 |
| Akhter, 2011 (363) | Rheumatol Int | Not Specified | 1980-Current |
| Usenbo, 2015 (364) | PLoS One | Systematic Review & Meta-Analysis | 1975-2014 |
| Almoallim, 2014 (365) | Saudi Med J | Systematic Review | 1984-2014 |
| Stolwijk, 2016 (366) | Arthritis Care Res (Hoboken) | Systematic Review & Meta-Regression | 1975-2014 |
| Mustafa, 2014 (367) | Clin Rheumatol | Not Specified | Inception-2013 |
| Osio-Salido, 2010 (368) | Lupus | Not Specified | Inception-2009 |
| Rafeey, 2015 (369) | Korean J Pediatr | Not Specified | 1980-2013 |
| Kadir, 2008 (370) | Public Health | Not Specified | Unmentioned |
| Chippaux, 2008 (371) | Acta Trop | Not Specified | 1979-Currnet |
| Othman, 2010 (372) | BMC Public Health | Systematic Review | 1997-2007 |
| Golshan, 2013 (373) | J Public Health (Oxf) | Systematic Review | 1970-2011 |
| Tapp, 2008 (374) | Confl Health | Systematic Review | Inception-2008 |
| Fehling, 2016 (375) | East Mediterr Health J | Systematic Review | Unmentioned |
| Cheng, 2011 (376) | Osteoporos Int | Systematic Review | Inception-2009 |
| Kulczycki, 2011 (377) | Violence Against Women | Systematic Review | Inception-Current |
| Ali, 2015 (378) | Trauma Violence Abuse | Systematic Review | 1985-2011 |
| Roman, 2013 (379) | Fam Pract | Systematic Review | 2002-2012 |
| Boy, 2008 (380) | Violence Against Women | Not Specified | Unmentioned |
| Majzoub, 2015 (381) | Urol Ann | Not Specified | 2003-2014 |
| McAlpine, 2016 (382) | BMC Int Health Hum Rights | Systematic Review | 2000-2014 |
| Mansuri, 2015 (383) | Saudi Med J | Systematic Review | 1990-2015 |
| Puvanachandra, 2012 (384) | Traffic Inj Prev | Not Specified | Unmentioned |
| Abou-Abbass, 2016 (385) | Medicine (Baltimore) | Systematic Review | Inception-2016 |
| Cripps, 2011 (386) | Spinal Cord | Not Specified | Unmentioned- Unmentioned |
| Hillis, 2016 (387) | Pediatrics | Systematic Review | 2000-2015 |

1. AlMarri TS, Oei TP. Alcohol and substance use in the Arabian Gulf region: a review. International journal of psychology : Journal international de psychologie. 2009;44(3):222-33.

2. Popova S, Lange S, Probst C, Shield K, Kraicer-Melamed H, Ferreira-Borges C, et al. Actual and predicted prevalence of alcohol consumption during pregnancy in the WHO African Region. Tropical medicine & international health : TM & IH. 2016;21(10):1209-39.

3. Ghandour L, Chalak A, El-Aily A, Yassin N, Nakkash R, Tauk M, et al. Alcohol consumption in the Arab region: What do we know, why does it matter, and what are the policy implications for youth harm reduction? The International journal on drug policy. 2016;28:10-33.

4. Francis JM, Grosskurth H, Changalucha J, Kapiga SH, Weiss HA. Systematic review and meta-analysis: prevalence of alcohol use among young people in eastern Africa. Tropical medicine & international health : TM & IH. 2014;19(4):476-88.

5. John LJ, Muttappallymyalil J. Dokha: an emerging public health issue as a form of tobacco smoking in the middle East. Asian Pacific journal of cancer prevention : APJCP. 2013;14(12):7065-7.

6. Degenhardt L, Bucello C, Calabria B, Nelson P, Roberts A, Hall W, et al. What data are available on the extent of illicit drug use and dependence globally? Results of four systematic reviews. Drug Alcohol Depend. 2011;117(2):85-101.

7. Degenhardt L, Charlson F, Mathers B, Hall WD, Flaxman AD, Johns N, et al. The global epidemiology and burden of opioid dependence: results from the global burden of disease 2010 study. Addiction. 2014;109(8):1320-33.

8. Alsanosy RM. Smokeless tobacco (shammah) in Saudi Arabia: a review of its pattern of use, prevalence, and potential role in oral cancer. Asian Pacific journal of cancer prevention : APJCP. 2014;15(16):6477-83.

9. Harbour C. Smoking and normative influence among Egyptian youth: a review of the literature. Eastern Mediterranean health journal = La revue de sante de la Mediterranee orientale = al-Majallah al-sihhiyah li-sharq al-mutawassit. 2011;17(4):349-55.

10. Almutairi KM. Smoking among Saudi students: a review of risk factors and early intentions of smoking. J Community Health. 2014;39(5):901-7.

11. Shah N, Siddiqui S. An overview of smoking practices in Pakistan. Pak J Med Sci. 2015;31(2):467-70.

12. Abdullah AS, Stillman FA, Yang L, Luo H, Zhang Z, Samet JM. Tobacco use and smoking cessation practices among physicians in developing countries: a literature review (1987-2010). International journal of environmental research and public health. 2013;11(1):429-55.

13. Kassim S, Jawad M, Croucher R, Akl EA. The Epidemiology of Tobacco Use among Khat Users: A Systematic Review. BioMed research international. 2015;2015:313692.

14. Akl EA, Gunukula SK, Aleem S, Obeid R, Jaoude PA, Honeine R, et al. The prevalence of waterpipe tobacco smoking among the general and specific populations: a systematic review. BMC Public Health. 2011;11:244.

15. Akl EA, Jawad M, Lam WY, Co CN, Obeid R, Irani J. Motives, beliefs and attitudes towards waterpipe tobacco smoking: a systematic review. Harm reduction journal. 2013;10:12.

16. Hassen K, Abdulahi M, Dejene T, Wolde M, Sudhakar M. Khat as a risk factor for hypertension: A systematic review. JBI library of systematic reviews. 2012;10(44):2882-905.

17. El-Zaemey S, Schuz J, Leon ME. Qat Chewing and Risk of Potentially Malignant and Malignant Oral Disorders: A Systematic Review. The international journal of occupational and environmental medicine. 2015;6(3):129-43.

18. Kakde S, Bhopal RS, Jones CM. A systematic review on the social context of smokeless tobacco use in the South Asian population: implications for public health. Public Health. 2012;126(8):635-45.

19. Katelaris CH, Lee BW, Potter PC, Maspero JF, Cingi C, Lopatin A, et al. Prevalence and diversity of allergic rhinitis in regions of the world beyond Europe and North America. Clinical and experimental allergy : journal of the British Society for Allergy and Clinical Immunology. 2012;42(2):186-207.

20. Aldrees AM. Lateral cephalometric norms for Saudi adults: A meta-analysis. The Saudi dental journal. 2011;23(1):3-7.

21. Sabbagh HJ, Mossey PA, Innes NP. Prevalence of orofacial clefts in Saudi Arabia and neighboring countries: A systematic review. The Saudi dental journal. 2012;24(1):3-10.

22. Yammine K. Clinical prevalence of palmaris longus agenesis: a systematic review and meta-analysis. Clinical anatomy (New York, NY). 2013;26(6):709-18.

23. Ameh OI, Kengne AP, Jayne D, Bello AK, Hodkinson B, Gcelu A, et al. Standard of treatment and outcomes of adults with lupus nephritis in Africa: a systematic review. Lupus. 2016;25(11):1269-77.

24. Lip GY, Brechin CM, Lane DA. The global burden of atrial fibrillation and stroke: a systematic review of the epidemiology of atrial fibrillation in regions outside North America and Europe. Chest. 2012;142(6):1489-98.

25. Alshaikh MK, Filippidis FT, Baldove JP, Majeed A, Rawaf S. Women in Saudi Arabia and the Prevalence of Cardiovascular Risk Factors: A Systematic Review. J Environ Public Health. 2016;2016:7479357.

26. Deek H, Newton P, Inglis S, Kabbani S, Noureddine S, Macdonald PS, et al. Heart health in Lebanon and considerations for addressing the burden of cardiovascular disease. Collegian. 2015;22(3):333-9.

27. Benamer HT, Grosset D. Stroke in Arab countries: a systematic literature review. J Neurol Sci. 2009;284(1):18-23.

28. Tran J, Mirzaei M, Anderson L, Leeder SR. The epidemiology of stroke in the Middle East and North Africa. J Neurol Sci. 2010;295(1):38-40.

29. Behrouz R, Powers CJ. Epidemiology of classical risk factors in stroke patients in the Middle East. European journal of neurology : the official journal of the European Federation of Neurological Societies. 2016;23(2):262-9.

30. Khan AA, Zafar SN. Venous thromboembolism in Pakistan: a neglected research agenda. Journal of thrombosis and thrombolysis. 2013;35(2):234-42.

31. Yamamoto SS, Phalkey R, Malik AA. A systematic review of air pollution as a risk factor for cardiovascular disease in South Asia: limited evidence from India and Pakistan. International journal of hygiene and environmental health. 2014;217(2):133-44.

32. Nasser Z, Salameh P, Nasser W, Abou Abbas L, Elias E, Leveque A. Outdoor particulate matter (PM) and associated cardiovascular diseases in the Middle East. International journal of occupational medicine and environmental health. 2015;28(4):641-61.

33. Gonzalez JV, Barboza AG, Vazquez FJ, Gandara E. Prevalence and Geographical Variation of Prothrombin G20210A Mutation in Patients with Cerebral Vein Thrombosis: A Systematic Review and Meta-Analysis. PLoS One. 2016;11(3):e0151607.

34. Akeroyd JM, Chan WJ, Kamal AK, Palaniappan L, Virani SS. Adherence to cardiovascular medications in the South Asian population: A systematic review of current evidence and future directions. World J Cardiol. 2015;7(12):938-47.

35. Donnelly TT, Al Suwaidi JM, Al-Qahtani A, Asaad N, Qader NA, Singh R, et al. Depression in Cardiovascular Patients in Middle Eastern Populations: A Literature Review. Journal of immigrant and minority health / Center for Minority Public Health. 2015;17(4):1259-76.

36. Barakat H, Barakat H, Baaj MK. CVD and obesity in transitional Syria: a perspective from the Middle East. Vascular health and risk management. 2012;8:145-50.

37. Raza Q, Doak CM, Khan A, Nicolaou M, Seidell JC. Obesity and cardiovascular disease risk factors among the indigenous and immigrant Pakistani population: a systematic review. Obesity facts. 2013;6(6):523-35.

38. Aljefree N, Ahmed F. Association between dietary pattern and risk of cardiovascular disease among adults in the Middle East and North Africa region: a systematic review. Food Nutr Res. 2015;59:27486.

39. Mehio Sibai A, Nasreddine L, Mokdad AH, Adra N, Tabet M, Hwalla N. Nutrition transition and cardiovascular disease risk factors in Middle East and North Africa countries: reviewing the evidence. Ann Nutr Metab. 2010;57(3):193-203.

40. Nasreddine L, Naja FA, Sibai AM, Helou K, Adra N, Hwalla N. Trends in nutritional intakes and nutrition-related cardiovascular disease risk factors in Lebanon: the need for immediate action. Le Journal medical libanais The Lebanese medical journal. 2014;62(2):83-91.

41. Smythe T, Kuper H, Macleod D, Foster A, Lavy C. Birth prevalence of congenital talipes equinovarus in low- and middle-income countries: a systematic review and meta-analysis. Tropical medicine & international health : TM & IH. 2016;0.

42. Vynnycky E, Adams EJ, Cutts FT, Reef SE, Navar AM, Simons E, et al. Using Seroprevalence and Immunisation Coverage Data to Estimate the Global Burden of Congenital Rubella Syndrome, 1996-2010: A Systematic Review. PLoS One. 2016;11(3):e0149160.

43. Deckers IA, McLean S, Linssen S, Mommers M, van Schayck CP, Sheikh A. Investigating international time trends in the incidence and prevalence of atopic eczema 1990-2010: a systematic review of epidemiological studies. PLoS One. 2012;7(7):e39803.

44. Jayawardena R, Ranasinghe P, Byrne NM, Soares MJ, Katulanda P, Hills AP. Prevalence and trends of the diabetes epidemic in South Asia: a systematic review and meta-analysis. BMC Public Health. 2012;12:380.

45. Bos M, Agyemang C. Prevalence and complications of diabetes mellitus in Northern Africa, a systematic review. BMC Public Health. 2013;13:387.

46. Zabetian A, Keli HM, Echouffo-Tcheugui JB, Narayan KM, Ali MK. Diabetes in the Middle East and North Africa. Diabetes Res Clin Pract. 2013;101(2):106-22.

47. Lamri L, Gripiotis E, Ferrario A. Diabetes in Algeria and challenges for health policy: a literature review of prevalence, cost, management and outcomes of diabetes and its complications. Globalization and health. 2014;10:11.

48. NCD Risk Factor Collaboration. Worldwide trends in diabetes since 1980: a pooled analysis of 751 population-based studies with 4.4 million participants. Lancet. 2016;387(10027):1513-30.

49. Mbanya V, Hussain A, Kengne AP. Application and applicability of non-invasive risk models for predicting undiagnosed prevalent diabetes in Africa: A systematic literature search. Primary care diabetes. 2015;9(5):317-29.

50. Meo SA. Prevalence and future prediction of type 2 diabetes mellitus in the Kingdom of Saudi Arabia: A systematic review of published studies. JPMA The Journal of the Pakistan Medical Association. 2016;66(6):722-5.

51. Zayed H. Epidemiology of diabetic ketoacidosis in Arab patients with type 1 diabetes: a systematic review. International journal of clinical practice. 2016;70(3):186-95.

52. Usher-Smith JA, Thompson M, Ercole A, Walter FM. Variation between countries in the frequency of diabetic ketoacidosis at first presentation of type 1 diabetes in children: a systematic review. Diabetologia. 2012;55(11):2878-94.

53. Danaei G, Finucane MM, Lu Y, Singh GM, Cowan MJ, Paciorek CJ, et al. National, regional, and global trends in fasting plasma glucose and diabetes prevalence since 1980: systematic analysis of health examination surveys and epidemiological studies with 370 country-years and 2.7 million participants. Lancet. 2011;378(9785):31-40.

54. Alhyas L, McKay A, Majeed A. Prevalence of type 2 diabetes in the States of the co-operation council for the Arab States of the Gulf: a systematic review. PLoS One. 2012;7(8):e40948.

55. Meo SA, Zia I, Bukhari IA, Arain SA. Type 2 diabetes mellitus in Pakistan: Current prevalence and future forecast. JPMA The Journal of the Pakistan Medical Association. 2016;66(12):1637-42.

56. Alkabab YM, Al-Abdely HM, Heysell SK. Diabetes-related tuberculosis in the Middle East: an urgent need for regional research. International journal of infectious diseases : IJID : official publication of the International Society for Infectious Diseases. 2015;40:64-70.

57. Hassan A, Meo SA. Diabetes during Ramadan: underestimated, under-investigated, needs more attention. European review for medical and pharmacological sciences. 2014;18(22):3528-33.

58. Yako YY, Guewo-Fokeng M, Balti EV, Bouatia-Naji N, Matsha TE, Sobngwi E, et al. Genetic risk of type 2 diabetes in populations of the African continent: A systematic review and meta-analyses. Diabetes Res Clin Pract. 2016;114:136-50.

59. Hamzeh AR, Nair P, Al Ali MT. The profile of HLA-DRB1 alleles in Arabs with type 1 diabetes; meta-analyses. Hla. 2016;87(1):25-30.

60. Hamzeh AR, Nair P, Al-Khaja N, Al Ali MT. Association of HLA-DQA1 and -DQB1 alleles with type I diabetes in Arabs: a meta-analyses. Tissue antigens. 2015;86(1):21-7.

61. Alharbi NS, Almutari R, Jones S, Al-Daghri N, Khunti K, de Lusignan S. Trends in the prevalence of type 2 diabetes mellitus and obesity in the Arabian Gulf States: systematic review and meta-analysis. Diabetes Res Clin Pract. 2014;106(2):e30-3.

62. Al-Khudairy L, Stranges S, Kumar S, Al-Daghri N, Rees K. Dietary factors and type 2 diabetes in the Middle East: what is the evidence for an association?--a systematic review. Nutrients. 2013;5(10):3871-97.

63. Fleming E, Gillibrand W. An exploration of culture, diabetes, and nursing in the South Asian community: a metasynthesis of qualitative studies. Journal of transcultural nursing : official journal of the Transcultural Nursing Society / Transcultural Nursing Society. 2009;20(2):146-55.

64. Motlagh B, O'Donnell M, Yusuf S. Prevalence of cardiovascular risk factors in the Middle East: a systematic review. European journal of cardiovascular prevention and rehabilitation : official journal of the European Society of Cardiology, Working Groups on Epidemiology & Prevention and Cardiac Rehabilitation and Exercise Physiology. 2009;16(3):268-80.

65. Khader YS, Abdelrahman M, Abdo N, Al-Sharif M, Elbetieha A, Bakir H, et al. Climate change and health in the Eastern Mediterranean countries: a systematic review. Reviews on environmental health. 2015;30(3):163-81.

66. DeNicola E, Aburizaiza OS, Siddique A, Khwaja H, Carpenter DO. Climate Change and Water Scarcity: The Case of Saudi Arabia. Annals of global health. 2015;81(3):342-53.

67. Singh P, Arora S, Singh A, Strand TA, Makharia GK. Prevalence of celiac disease in Asia: A systematic review and meta-analysis. Journal of gastroenterology and hepatology. 2016;31(6):1095-101.

68. Gautret P, Benkouiten S, Sridhar S, Al-Tawfiq JA, Memish ZA. Diarrhea at the Hajj and Umrah. Travel medicine and infectious disease. 2015;13(2):159-66.

69. Olusanya BO, Osibanjo FB, Slusher TM. Risk factors for severe neonatal hyperbilirubinemia in low and middle-income countries: a systematic review and meta-analysis. PLoS One. 2015;10(2):e0117229.

70. Molodecky NA, Soon IS, Rabi DM, Ghali WA, Ferris M, Chernoff G, et al. Increasing incidence and prevalence of the inflammatory bowel diseases with time, based on systematic review. Gastroenterology. 2012;142(1):46-54.e42; quiz e30.

71. Devanarayana NM, Rajindrajith S, Pathmeswaran A, Abegunasekara C, Gunawardena NK, Benninga MA. Epidemiology of irritable bowel syndrome in children and adolescents in Asia. Journal of pediatric gastroenterology and nutrition. 2015;60(6):792-8.

72. Sperber AD, Dumitrascu D, Fukudo S, Gerson C, Ghoshal UC, Gwee KA, et al. The global prevalence of IBS in adults remains elusive due to the heterogeneity of studies: a Rome Foundation working team literature review. Gut. 2016;0.

73. Ashtari S, Pourhoseingholi MA, Zali MR. Non-alcohol fatty liver disease in Asia: Prevention and planning. World journal of hepatology. 2015;7(13):1788-96.

74. Jiang J, Jiang B, Parashar U, Nguyen T, Bines J, Patel MM. Childhood intussusception: a literature review. PLoS One. 2013;8(7):e68482.

75. de Menthon M, Lavalley MP, Maldini C, Guillevin L, Mahr A. HLA-B51/B5 and the risk of Behcet's disease: a systematic review and meta-analysis of case-control genetic association studies. Arthritis and rheumatism. 2009;61(10):1287-96.

76. Romdhane L, Kefi R, Azaiez H, Ben Halim N, Dellagi K, Abdelhak S. Founder mutations in Tunisia: implications for diagnosis in North Africa and Middle East. Orphanet journal of rare diseases. 2012;7:52.

77. Doss CG, Alasmar DR, Bux RI, Sneha P, Bakhsh FD, Al-Azwani I, et al. Genetic Epidemiology of Glucose-6-Dehydrogenase Deficiency in the Arab World. Scientific reports. 2016;6:37284.

78. Romdhane L, Abdelhak S. Genetic diseases in the Tunisian population. American journal of medical genetics Part A. 2011;155(1):238-67.

79. Sabahelzain MM, Hamamy H. The ethnic distribution of sickle cell disease in Sudan. The Pan African medical journal. 2014;18:13.

80. Habeb AM. Frequency and spectrum of Wolcott-Rallison syndrome in Saudi Arabia: a systematic review. The Libyan journal of medicine. 2013;8:21137.

81. Mahdieh N, Rabbani B. Beta thalassemia in 31,734 cases with HBB gene mutations: Pathogenic and structural analysis of the common mutations; Iran as the crossroads of the Middle East. Blood reviews. 2016;30(6):493-508.

82. Bader LA, Elewa H. The Impact of Genetic and Non-Genetic Factors on Warfarin Dose Prediction in MENA Region: A Systematic Review. PLoS One. 2016;11(12):e0168732.

83. Gaikwad T, Ghosh K, Shetty S. VKORC1 and CYP2C9 genotype distribution in Asian countries. Thrombosis research. 2014;134(3):537-44.

84. Inhorn MC, Patrizio P. Infertility around the globe: new thinking on gender, reproductive technologies and global movements in the 21st century. Human reproduction update. 2015;21(4):411-26.

85. Mandil A, Chaaya M, Saab D. Health status, epidemiological profile and prospects: Eastern Mediterranean Region. International journal of epidemiology. 2013;42(2):616-26.

86. Tajvar M, Fletcher A, Grundy E, Arab M. Social support and health of older people in Middle Eastern countries: a systematic review. Australas J Ageing. 2013;32(2):71-8.

87. Benova L, Campbell OM, Ploubidis GB. Socio-economic gradients in maternal and child health-seeking behaviours in egypt: systematic literature review and evidence synthesis. PLoS One. 2014;9(3):e93032.

88. Salmon-Rousseau A, Piednoir E, Cattoir V, de La Blanchardiere A. Hajj-associated infections. Medecine et maladies infectieuses. 2016;46(7):346-54.

89. Tosson AM, Speer CP. Microbial pathogens causative of neonatal sepsis in Arabic countries. The journal of maternal-fetal & neonatal medicine : the official journal of the European Association of Perinatal Medicine, the Federation of Asia and Oceania Perinatal Societies, the International Society of Perinatal Obstet. 2011;24(8):990-4.

90. Gautret P, Steffen R. Communicable diseases as health risks at mass gatherings other than Hajj: what is the evidence? International journal of infectious diseases : IJID : official publication of the International Society for Infectious Diseases. 2016;47:46-52.

91. Prasad N, Murdoch DR, Reyburn H, Crump JA. Etiology of Severe Febrile Illness in Low- and Middle-Income Countries: A Systematic Review. PLoS One. 2015;10(6):e0127962.

92. DeAntonio R, Yarzabal JP, Cruz JP, Schmidt JE, Kleijnen J. Epidemiology of otitis media in children from developing countries: A systematic review. International journal of pediatric otorhinolaryngology. 2016;85:65-74.

93. Musallam, II, Abo-Shehada MN, Hegazy YM, Holt HR, Guitian FJ. Systematic review of brucellosis in the Middle East: disease frequency in ruminants and humans and risk factors for human infection. Epidemiology and infection. 2016;144(4):671-85.

94. Naseer M, Jamali T. Epidemiology, determinants and dynamics of cholera in Pakistan: gaps and prospects for future research. Journal of the College of Physicians and Surgeons--Pakistan : JCPSP. 2014;24(11):855-60.

95. Buckle GC, Walker CL, Black RE. Typhoid fever and paratyphoid fever: Systematic review to estimate global morbidity and mortality for 2010. Journal of global health. 2012;2(1):010401.

96. Azmatullah A, Qamar FN, Thaver D, Zaidi AK, Bhutta ZA. Systematic review of the global epidemiology, clinical and laboratory profile of enteric fever. Journal of global health. 2015;5(2):020407.

97. Coffin LS, Newberry A, Hagan H, Cleland CM, Des Jarlais DC, Perlman DC. Syphilis in drug users in low and middle income countries. The International journal on drug policy. 2010;21(1):20-7.

98. Kenyon C, Colebunders R, Crucitti T. The global epidemiology of bacterial vaginosis: a systematic review. Am J Obstet Gynecol. 2013;209(6):505-23.

99. Manenzhe RI, Zar HJ, Nicol MP, Kaba M. The spread of carbapenemase-producing bacteria in Africa: a systematic review. The Journal of antimicrobial chemotherapy. 2015;70(1):23-40.

100. Vanderburg S, Rubach MP, Halliday JE, Cleaveland S, Reddy EA, Crump JA. Epidemiology of Coxiella burnetii infection in Africa: a OneHealth systematic review. PLoS neglected tropical diseases. 2014;8(4):e2787.

101. Steer AC, Law I, Matatolu L, Beall BW, Carapetis JR. Global emm type distribution of group A streptococci: systematic review and implications for vaccine development. The Lancet Infectious diseases. 2009;9(10):611-6.

102. Khedmat H, Karbasi-Afshar R, Agah S, Taheri S. Helicobacter pylori Infection in the general population: A Middle Eastern perspective. Caspian J Intern Med. 2013;4(4):745-53.

103. Eshraghian A. Epidemiology of Helicobacter pylori infection among the healthy population in Iran and countries of the Eastern Mediterranean Region: a systematic review of prevalence and risk factors. World journal of gastroenterology : WJG. 2014;20(46):17618-25.

104. Dean AS, Crump L, Greter H, Schelling E, Zinsstag J. Global burden of human brucellosis: a systematic review of disease frequency. PLoS neglected tropical diseases. 2012;6(10):e1865.

105. Shibl A, Memish Z, Pelton S. Epidemiology of invasive pneumococcal disease in the Arabian Peninsula and Egypt. International journal of antimicrobial agents. 2009;33(5):410.e1-9.

106. Hung IF, Tantawichien T, Tsai YH, Patil S, Zotomayor R. Regional epidemiology of invasive pneumococcal disease in Asian adults: epidemiology, disease burden, serotype distribution, and antimicrobial resistance patterns and prevention. International journal of infectious diseases : IJID : official publication of the International Society for Infectious Diseases. 2013;17(6):e364-73.

107. Fares A. Seasonality of tuberculosis. Journal of global infectious diseases. 2011;3(1):46-55.

108. Horton KC, MacPherson P, Houben RM, White RG, Corbett EL. Sex Differences in Tuberculosis Burden and Notifications in Low- and Middle-Income Countries: A Systematic Review and Meta-analysis. PLoS medicine. 2016;13(9):e1002119.

109. Ghenghesh KS, Rahouma A, Zorgani A, Tawil K, Al Tomi A, Franka E. Aeromonas in Arab countries: 1995-2014. Comp Immunol Microbiol Infect Dis. 2015;42:8-14.

110. Tansarli GS, Poulikakos P, Kapaskelis A, Falagas ME. Proportion of extended-spectrum beta-lactamase (ESBL)-producing isolates among Enterobacteriaceae in Africa: evaluation of the evidence--systematic review. The Journal of antimicrobial chemotherapy. 2014;69(5):1177-84.

111. Areeshi MY, Bisht SC, Mandal RK, Haque S. Prevalence of drug resistance in clinical isolates of tuberculosis from GCC: a literature review from January 2002 to March 2013. Journal of infection in developing countries. 2014;8(9):1137-47.

112. Sangare SA, Maiga AI, Guindo I, Maiga A, Camara N, Savadogo S, et al. Prevalence of extended-spectrum beta-lactamase-producing Enterobacteriaceae isolated from blood cultures in Africa. Medecine et maladies infectieuses. 2015;45(9):374-82.

113. Abdulgader SM, Shittu AO, Nicol MP, Kaba M. Molecular epidemiology of Methicillin-resistant Staphylococcus aureus in Africa: a systematic review. Frontiers in microbiology. 2015;6:348.

114. Mamishi S, Moradkhani S, Mahmoudi S, Hosseinpour-Sadeghi R, Pourakbari B. Penicillin-Resistant trend of Streptococcus pneumoniae in Asia: A systematic review. Iran J Microbiol. 2014;6(4):198-210.

115. Jaiswal N, Singh M, Das RR, Jindal I, Agarwal A, Thumburu KK, et al. Distribution of serotypes, vaccine coverage, and antimicrobial susceptibility pattern of Streptococcus pneumoniae in children living in SAARC countries: a systematic review. PLoS One. 2014;9(9):e108617.

116. Gu B, Zhou M, Ke X, Pan S, Cao Y, Huang Y, et al. Comparison of resistance to third-generation cephalosporins in Shigella between Europe-America and Asia-Africa from 1998 to 2012. Epidemiology and infection. 2015;143(13):2687-99.

117. Kahsay AG, Muthupandian S. A review on Sero diversity and antimicrobial resistance patterns of Shigella species in Africa, Asia and South America, 2001-2014. BMC research notes. 2016;9(1):422.

118. Berrazeg M, Diene S, Medjahed L, Parola P, Drissi M, Raoult D, et al. New Delhi Metallo-beta-lactamase around the world: an eReview using Google Maps. Euro Surveill. 2014;19(20).

119. Velayati AA, Rahideh S, Nezhad ZD, Farnia P, Mirsaeidi M. Nontuberculous mycobacteria in Middle East: Current situation and future challenges. Int J Mycobacteriol. 2015;4(1):7-17.

120. Al-Tawfiq JA, Hinedi K, Memish ZA. Systematic review of the prevalence of Mycobacterium tuberculosis resistance in Saudi Arabia. Journal of chemotherapy (Florence, Italy). 2015;27(6):378-82.

121. Chakrabarti A, Chatterjee SS, Das A, Shivaprakash MR. Invasive aspergillosis in developing countries. Medical mycology. 2011;49:S35-47.

122. van de Sande WW. Global burden of human mycetoma: a systematic review and meta-analysis. PLoS neglected tropical diseases. 2013;7(11):e2550.

123. Romani L, Steer AC, Whitfeld MJ, Kaldor JM. Prevalence of scabies and impetigo worldwide: a systematic review. The Lancet Infectious diseases. 2015;15(8):960-7.

124. Araj GF, Mourad Y. Hydatid disease: the Lebanese contribution. Le Journal medical libanais The Lebanese medical journal. 2014;62(4):217-26.

125. D'Acremont V, Lengeler C, Genton B. Reduction in the proportion of fevers associated with Plasmodium falciparum parasitaemia in Africa: a systematic review. Malaria journal. 2010;9:240.

126. Rahimi BA, Thakkinstian A, White NJ, Sirivichayakul C, Dondorp AM, Chokejindachai W. Severe vivax malaria: a systematic review and meta-analysis of clinical studies since 1900. Malaria journal. 2014;13:481.

127. Coleman M, Al-Zahrani MH, Coleman M, Hemingway J, Omar A, Stanton MC, et al. A country on the verge of malaria elimination--the Kingdom of Saudi Arabia. PLoS One. 2014;9(9):e105980.

128. Pappas G, Roussos N, Falagas ME. Toxoplasmosis snapshots: global status of Toxoplasma gondii seroprevalence and implications for pregnancy and congenital toxoplasmosis. Int J Parasitol. 2009;39(12):1385-94.

129. Alsammani MA. Sero-epidemiology and risk factors for Toxoplasma gondii among pregnant women in Arab and African countries. Journal of parasitic diseases : official organ of the Indian Society for Parasitology. 2016;40(3):569-79.

130. Al-Salem W, Herricks JR, Hotez PJ. A review of visceral leishmaniasis during the conflict in South Sudan and the consequences for East African countries. Parasites & vectors. 2016;9:460.

131. Youssef FG, Adib I, Riddle MS, Schlett CD. A review of cryptosporidiosis in Egypt. Journal of the Egyptian Society of Parasitology. 2008;38(1):9-28.

132. Roberts T, Gravett CA, Velu PP, Theodoratou E, Wagner TA, Zhang JS, et al. Epidemiology and aetiology of maternal parasitic infections in low- and middle-income countries. Journal of global health. 2011;1(2):189-200.

133. Chanda E, Govere JM, Macdonald MB, Lako RL, Haque U, Baba SP, et al. Integrated vector management: a critical strategy for combating vector-borne diseases in South Sudan. Malaria journal. 2013;12:369.

134. Ansarie M, Kasmani A. Community acquired pneumonia in Pakistan: an analysis on the literature published between 2003 and 2013. JPMA The Journal of the Pakistan Medical Association. 2014;64(12):1405-9.

135. DeAntonio R, Yarzabal JP, Cruz JP, Schmidt JE, Kleijnen J. Epidemiology of community-acquired pneumonia and implications for vaccination of children living in developing and newly industrialized countries: A systematic literature review. Hum Vaccin Immunother. 2016;12(9):2422-40.

136. Alqahtani AS, Rashid H, Heywood AE. Vaccinations against respiratory tract infections at Hajj. Clinical microbiology and infection : the official publication of the European Society of Clinical Microbiology and Infectious Diseases. 2015;21(2):115-27.

137. Gasim GI, Bella A, Adam I. Schistosomiasis, hepatitis B and hepatitis C co-infection. Virology journal. 2015;12:19.

138. Van-Lume DS, Albuquerque Mde F, Souza AI, Domingues AL, Lopes EP, Morais CN, et al. Association between Schistosomiasis mansoni and hepatitis C: systematic review. Revista de saude publica. 2013;47(2):414-24.

139. Bosan A, Qureshi H, Bile KM, Ahmad I, Hafiz R. A review of hepatitis viral infections in Pakistan. JPMA The Journal of the Pakistan Medical Association. 2010;60(12):1045-58.

140. Azevedo TC, Zwahlen M, Rauch A, Egger M, Wandeler G. Hepatitis C in HIV-infected individuals: a systematic review and meta-analysis of estimated prevalence in Africa. Journal of the International AIDS Society. 2016;19(1):20711.

141. Schweitzer A, Horn J, Mikolajczyk RT, Krause G, Ott JJ. Estimations of worldwide prevalence of chronic hepatitis B virus infection: a systematic review of data published between 1965 and 2013. Lancet. 2015;386(10003):1546-55.

142. Ali M, Idrees M, Ali L, Hussain A, Ur Rehman I, Saleem S, et al. Hepatitis B virus in Pakistan: a systematic review of prevalence, risk factors, awareness status and genotypes. Virology journal. 2011;8:102.

143. Ott JJ, Stevens GA, Wiersma ST. The risk of perinatal hepatitis B virus transmission: hepatitis B e antigen (HBeAg) prevalence estimates for all world regions. BMC infectious diseases. 2012;12:131.

144. Ezzikouri S, Pineau P, Benjelloun S. Hepatitis B virus in the Maghreb region: from epidemiology to prospective research. Liver international : official journal of the International Association for the Study of the Liver. 2013;33(6):811-9.

145. Gasim GI. Hepatitis B virus in the Arab world: where do we stand? Arab J Gastroenterol. 2013;14(2):35-43.

146. Babanejad M, Izadi N, Najafi F, Alavian SM. The HBsAg Prevalence Among Blood Donors From Eastern Mediterranean and Middle Eastern Countries: A Systematic Review and Meta-Analysis. Hepatitis monthly. 2016;16(3):e35664.

147. Bajubair MA, Elrub AA, Bather G. Hepatic viral infections in Yemen between 2000--2005. Saudi Med J. 2008;29(6):871-4.

148. Ali SA, Donahue RM, Qureshi H, Vermund SH. Hepatitis B and hepatitis C in Pakistan: prevalence and risk factors. International journal of infectious diseases : IJID : official publication of the International Society for Infectious Diseases. 2009;13(1):9-19.

149. Lehman EM, Wilson ML. Epidemiology of hepatitis viruses among hepatocellular carcinoma cases and healthy people in Egypt: a systematic review and meta-analysis. International journal of cancer Journal international du cancer. 2009;124(3):690-7.

150. Nelson PK, Mathers BM, Cowie B, Hagan H, Des Jarlais D, Horyniak D, et al. Global epidemiology of hepatitis B and hepatitis C in people who inject drugs: results of systematic reviews. Lancet. 2011;378(9791):571-83.

151. Gasim GI, Murad IA, Adam I. Hepatitis B and C virus infections among pregnant women in Arab and African countries. Journal of infection in developing countries. 2013;7(8):566-78.

152. Bashour H, Muhjazi G. Hepatitis B and C in the Syrian Arab Republic: a review. Eastern Mediterranean health journal = La revue de sante de la Mediterranee orientale = al-Majallah al-sihhiyah li-sharq al-mutawassit. 2016;22(4):267-73.

153. Riaz M, Idrees M, Kanwal H, Kabir F. An overview of triple infection with hepatitis B, C and D viruses. Virology journal. 2011;8:368.

154. Raja NS, Janjua KA. Epidemiology of hepatitis C virus infection in Pakistan. Journal of microbiology, immunology, and infection = Wei mian yu gan ran za zhi. 2008;41(1):4-8.

155. Stern RK, Hagan H, Lelutiu-Weinberger C, Des Jarlais D, Scheinmann R, Strauss S, et al. The HCV Synthesis Project: scope, methodology, and preliminary results. BMC Med Res Methodol. 2008;8:62.

156. Umar M, Khaar HT, Khurram M, Hasan Z. Anti-HCV antibody positivity of various sections of Pakistani patients. Journal of the College of Physicians and Surgeons--Pakistan : JCPSP. 2009;19(11):737-41.

157. Waheed Y, Shafi T, Safi SZ, Qadri I. Hepatitis C virus in Pakistan: a systematic review of prevalence, genotypes and risk factors. World journal of gastroenterology : WJG. 2009;15(45):5647-53.

158. Attaullah S, Khan S, Ali I. Hepatitis C virus genotypes in Pakistan: a systemic review. Virology journal. 2011;8:433.

159. Sievert W, Altraif I, Razavi HA, Abdo A, Ahmed EA, Alomair A, et al. A systematic review of hepatitis C virus epidemiology in Asia, Australia and Egypt. Liver international : official journal of the International Association for the Study of the Liver. 2011;31:61-80.

160. Alavian SM, Aalaei-Andabili SH. Lack of Knowledge About Hepatitis C Infection Rates Among Patients With Inherited Coagulation Disorders in Countries Under the Eastern Mediterranean Region Office of WHO (EMRO): A Meta-Analysis. Hepatitis monthly. 2012;12(4):244-52.

161. Ramia S, Melhem NM, Kreidieh K. Hepatitis C virus infection in the Middle East and North Africa "MENA" region: injecting drug users (IDUs) is an under-investigated population. Infection. 2012;40(1):1-10.

162. Karoney MJ, Siika AM. Hepatitis C virus (HCV) infection in Africa: a review. The Pan African medical journal. 2013;14:44.

163. Abozaid SM, Shoukri M, Al-Qahtani A, Al-Ahdal MN. Prevailing genotypes of hepatitis C virus in Saudi Arabia: a systematic analysis of evidence. Ann Saudi Med. 2013;33(1):1-5.

164. Ezzikouri S, Pineau P, Benjelloun S. Hepatitis C virus infection in the Maghreb region. Journal of medical virology. 2013;85(9):1542-9.

165. Mohd Hanafiah K, Groeger J, Flaxman AD, Wiersma ST. Global epidemiology of hepatitis C virus infection: new estimates of age-specific antibody to HCV seroprevalence. Hepatology. 2013;57(4):1333-42.

166. Mohamoud YA, Mumtaz GR, Riome S, Miller D, Abu-Raddad LJ. The epidemiology of hepatitis C virus in Egypt: a systematic review and data synthesis. BMC infectious diseases. 2013;13:288.

167. Reker C, Islam KM. Risk factors associated with high prevalence rates of hepatitis C infection in Egypt. International journal of infectious diseases : IJID : official publication of the International Society for Infectious Diseases. 2014;25:104-6.

168. Bruggmann P, Berg T, Ovrehus AL, Moreno C, Brandao Mello CE, Roudot-Thoraval F, et al. Historical epidemiology of hepatitis C virus (HCV) in selected countries. Journal of viral hepatitis. 2014;21:5-33.

169. Gower E, Estes C, Blach S, Razavi-Shearer K, Razavi H. Global epidemiology and genotype distribution of the hepatitis C virus infection. Journal of hepatology. 2014;61(1):S45-57.

170. El-Ghitany EM, Abdel Wahab MM, Abd El-Wahab EW, Hassouna S, Farghaly AG. A comprehensive hepatitis C virus risk factors meta-analysis (1989-2013): do they differ in Egypt? Liver international : official journal of the International Association for the Study of the Liver. 2015;35(2):489-501.

171. Chemaitelly H, Chaabna K, Abu-Raddad LJ. The Epidemiology of Hepatitis C Virus in the Fertile Crescent: Systematic Review and Meta-Analysis. PLoS One. 2015;10(8):e0135281.

172. Fadlalla FA, Mohamoud YA, Mumtaz GR, Abu-Raddad LJ. The epidemiology of hepatitis C virus in the Maghreb region: systematic review and meta-analyses. PLoS One. 2015;10(3):e0121873.

173. Riou J, Ait Ahmed M, Blake A, Vozlinsky S, Brichler S, Eholie S, et al. Hepatitis C virus seroprevalence in adults in Africa: a systematic review and meta-analysis. Journal of viral hepatitis. 2016;23(4):244-55.

174. Ghaderi-Zefrehi H, Gholami-Fesharaki M, Sharafi H, Sadeghi F, Alavian SM. The Distribution of Hepatitis C Virus Genotypes in Middle Eastern Countries: A Systematic Review and Meta-Analysis. Hepatitis monthly. 2016;16(9):e40357.

175. Sadeghi F, Salehi-Vaziri M, Almasi-Hashiani A, Gholami-Fesharaki M, Pakzad R, Alavian SM. Prevalence of Hepatitis C Virus Genotypes Among Patients in Countries of the Eastern Mediterranean Regional Office of WHO (EMRO): A Systematic Review and Meta-Analysis. Hepatitis monthly. 2016;16(4):e35558.

176. Chaabna K, Kouyoumjian SP, Abu-Raddad LJ. Hepatitis C Virus Epidemiology in Djibouti, Somalia, Sudan, and Yemen: Systematic Review and Meta-Analysis. PLoS One. 2016;11(2):e0149966.

177. Mohamoud YA, Riome S, Abu-Raddad LJ. Epidemiology of hepatitis C virus in the Arabian Gulf countries: Systematic review and meta-analysis of prevalence. International journal of infectious diseases : IJID : official publication of the International Society for Infectious Diseases. 2016;46:116-25.

178. Amini N, Alavian SM, Kabir A, Aalaei-Andabili SH, Saiedi Hosseini SY, Rizzetto M. Prevalence of hepatitis d in the eastern mediterranean region: systematic review and meta analysis. Hepatitis monthly. 2013;13(1):e8210.

179. Alavian SM, Haghbin H. Relative Importance of Hepatitis B and C Viruses in Hepatocellular Carcinoma in EMRO Countries and the Middle East: A Systematic Review. Hepatitis monthly. 2016;16(3):e35106.

180. de Martel C, Maucort-Boulch D, Plummer M, Franceschi S. World-wide relative contribution of hepatitis B and C viruses in hepatocellular carcinoma. Hepatology. 2015;62(4):1190-200.

181. Jacobsen KH, Wiersma ST. Hepatitis A virus seroprevalence by age and world region, 1990 and 2005. Vaccine. 2010;28(41):6653-7.

182. Itani T, Jacobsen KH, Nguyen T, Wiktor SZ. A new method for imputing country-level estimates of hepatitis A virus endemicity levels in the Eastern Mediterranean region. Vaccine. 2014;32(46):6067-74.

183. Yazbek S, Kreidieh K, Ramia S. Hepatitis E virus in the countries of the Middle East and North Africa region: an awareness of an infectious threat to blood safety. Infection. 2016;44(1):11-22.

184. Parashar UD, Burton A, Lanata C, Boschi-Pinto C, Shibuya K, Steele D, et al. Global mortality associated with rotavirus disease among children in 2004. The Journal of infectious diseases. 2009;200:S9-s15.

185. Khoury H, Ogilvie I, El Khoury AC, Duan Y, Goetghebeur MM. Burden of rotavirus gastroenteritis in the Middle Eastern and North African pediatric population. BMC infectious diseases. 2011;11:9.

186. Miles MG, Lewis KD, Kang G, Parashar UD, Steele AD. A systematic review of rotavirus strain diversity in India, Bangladesh, and Pakistan. Vaccine. 2012;30:A131-9.

187. Kawai K, O'Brien MA, Goveia MG, Mast TC, El Khoury AC. Burden of rotavirus gastroenteritis and distribution of rotavirus strains in Asia: a systematic review. Vaccine. 2012;30(7):1244-54.

188. Tate JE, Burton AH, Boschi-Pinto C, Steele AD, Duque J, Parashar UD. 2008 estimate of worldwide rotavirus-associated mortality in children younger than 5 years before the introduction of universal rotavirus vaccination programmes: a systematic review and meta-analysis. The Lancet Infectious diseases. 2012;12(2):136-41.

189. Jroundi I, Mahraoui C, Benmessaoud R, Moraleda C, Benjelloun B, Bassat Q. Knowledge gaps on paediatric respiratory infections in Morocco, Northern Africa. Arch Public Health. 2015;73(1):28.

190. Abubakar A, Malik M, Pebody RG, Elkholy AA, Khan W, Bellos A, et al. Burden of acute respiratory disease of epidemic and pandemic potential in the WHO Eastern Mediterranean Region: A literature review. Eastern Mediterranean health journal = La revue de sante de la Mediterranee orientale = al-Majallah al-sihhiyah li-sharq al-mutawassit. 2016;22(7):513-26.

191. Mathur MB, Patel RB, Gould M, Uyeki TM, Bhattacharya J, Xiao Y, et al. Seasonal patterns in human A (H5N1) virus infection: analysis of global cases. PLoS One. 2014;9(9):e106171.

192. Lai S, Qin Y, Cowling BJ, Ren X, Wardrop NA, Gilbert M, et al. Global epidemiology of avian influenza A H5N1 virus infection in humans, 1997-2015: a systematic review of individual case data. The Lancet Infectious diseases. 2016;16(7):e108-18.

193. Tricco AC, Lillie E, Soobiah C, Perrier L, Straus SE. Impact of H1N1 on socially disadvantaged populations: summary of a systematic review. Influenza and other respiratory viruses. 2013;7:54-8.

194. Alsolamy S. Middle East respiratory syndrome: knowledge to date. Critical care medicine. 2015;43(6):1283-90.

195. Gautret P, Benkouiten S, Al-Tawfiq JA, Memish ZA. Hajj-associated viral respiratory infections: A systematic review. Travel medicine and infectious disease. 2016;14(2):92-109.

196. Mathers BM, Degenhardt L, Phillips B, Wiessing L, Hickman M, Strathdee SA, et al. Global epidemiology of injecting drug use and HIV among people who inject drugs: a systematic review. Lancet. 2008;372(9651):1733-45.

197. Mathers BM, Degenhardt L, Ali H, Wiessing L, Hickman M, Mattick RP, et al. HIV prevention, treatment, and care services for people who inject drugs: a systematic review of global, regional, and national coverage. Lancet. 2010;375(9719):1014-28.

198. Abu-Raddad LJ, Hilmi N, Mumtaz G, Benkirane M, Akala FA, Riedner G, et al. Epidemiology of HIV infection in the Middle East and North Africa. Aids. 2010;24:S5-23.

199. Mumtaz G, Hilmi N, McFarland W, Kaplan RL, Akala FA, Semini I, et al. Are HIV epidemics among men who have sex with men emerging in the Middle East and North Africa?: a systematic review and data synthesis. PLoS medicine. 2010;8(8):e1000444.

200. Mumtaz G, Hilmi N, Akala FA, Semini I, Riedner G, Wilson D, et al. HIV-1 molecular epidemiology evidence and transmission patterns in the Middle East and North Africa. Sexually transmitted infections. 2011;87(2):101-6.

201. Kouyoumjian SP, Mumtaz GR, Hilmi N, Zidouh A, El Rhilani H, Alami K, et al. The epidemiology of HIV infection in Morocco: systematic review and data synthesis. International journal of STD & AIDS. 2013;24(7):507-16.

202. Mumtaz GR, Kouyoumjian SP, Hilmi N, Zidouh A, El Rhilani H, Alami K, et al. The distribution of new HIV infections by mode of exposure in Morocco. Sexually transmitted infections. 2013;89:iii49-56.

203. Singh S, Ambrosio M, Semini I, Tawil O, Saleem M, Imran M, et al. Revitalizing the HIV response in Pakistan: a systematic review and policy implications. The International journal on drug policy. 2014;25(1):26-33.

204. Mumtaz GR, Weiss HA, Thomas SL, Riome S, Setayesh H, Riedner G, et al. HIV among people who inject drugs in the Middle East and North Africa: systematic review and data synthesis. PLoS medicine. 2014;11(6):e1001663.

205. Poteat T, Scheim A, Xavier J, Reisner S, Baral S. Global Epidemiology of HIV Infection and Related Syndemics Affecting Transgender People. Journal of acquired immune deficiency syndromes (1999). 2016;72:S210-9.

206. Moradi A, Alammehrjerdi Z, Daneshmand R, Amini-Lari M, Zarghami M, Dolan K. HIV Responses in Arab States on the Southern Persian Gulf Border: The First Review. Iran J Psychiatry Behav Sci. 2016;10(3):e5392.

207. Lihana RW, Ssemwanga D, Abimiku A, Ndembi N. Update on HIV-1 diversity in Africa: a decade in review. AIDS reviews. 2012;14(2):83-100.

208. Abu-Raddad LJ, Schiffer JT, Ashley R, Mumtaz G, Alsallaq RA, Akala FA, et al. HSV-2 serology can be predictive of HIV epidemic potential and hidden sexual risk behavior in the Middle East and North Africa. Epidemics. 2010;2(4):173-82.

209. Caceres CF, Konda K, Segura ER, Lyerla R. Epidemiology of male same-sex behaviour and associated sexual health indicators in low- and middle-income countries: 2003-2007 estimates. Sexually transmitted infections. 2008;84:i49-i56.

210. Serbessa MK, Mariam DH, Kassa A, Alwan F, Kloos H. HIV/AIDS among pastoralists and refugees in north-east Africa: a neglected problem. African journal of AIDS research : AJAR. 2016;15(1):45-54.

211. Bruni L, Diaz M, Castellsague X, Ferrer E, Bosch FX, de Sanjose S. Cervical human papillomavirus prevalence in 5 continents: meta-analysis of 1 million women with normal cytological findings. The Journal of infectious diseases. 2010;202(12):1789-99.

212. Smith JS, Melendy A, Rana RK, Pimenta JM. Age-specific prevalence of infection with human papillomavirus in females: a global review. The Journal of adolescent health : official publication of the Society for Adolescent Medicine. 2008;43(4):S5-25, S.e1-41.

213. Ogembo RK, Gona PN, Seymour AJ, Park HS, Bain PA, Maranda L, et al. Prevalence of human papillomavirus genotypes among African women with normal cervical cytology and neoplasia: a systematic review and meta-analysis. PLoS One. 2015;10(4):e0122488.

214. Seoud M. Burden of human papillomavirus-related cervical disease in the extended middle East and north Africa-a comprehensive literature review. Journal of lower genital tract disease. 2012;16(2):106-20.

215. Alkaiyat A, Weiss MG. HIV in the Middle East and North Africa: priority, culture, and control. International journal of public health. 2013;58(6):927-37.

216. Furuya-Kanamori L, Liang S, Milinovich G, Soares Magalhaes RJ, Clements AC, Hu W, et al. Co-distribution and co-infection of chikungunya and dengue viruses. BMC infectious diseases. 2016;16:84.

217. Alhaeli A, Bahkali S, Ali A, Househ MS, El-Metwally AA. The epidemiology of Dengue fever in Saudi Arabia: A systematic review. J Infect Public Health. 2016;9(2):117-24.

218. Humphrey JM, Cleton NB, Reusken CB, Glesby MJ, Koopmans MP, Abu-Raddad LJ. Dengue in the Middle East and North Africa: A Systematic Review. PLoS neglected tropical diseases. 2016;10(12):e0005194.

219. Razavi SM, Saeednejad M, Salamati P. Vaccination in Hajj: An Overview of the Recent Findings. International journal of preventive medicine. 2016;7:129.

220. Ghenghesh KS, Rahouma A, Tawil K, Zorgani A, Franka E. Antimicrobial resistance in Libya: 1970-2011. The Libyan journal of medicine. 2013;8:1-8.

221. Tanios CY, Abou-Saleh MT, Karam AN, Salamoun MM, Mneimneh ZN, Karam EG. The epidemiology of anxiety disorders in the Arab world: a review. Journal of anxiety disorders. 2009;23(4):409-19.

222. Elzubeir MA, Elzubeir KE, Magzoub ME. Stress and coping strategies among Arab medical students: towards a research agenda. Education for health (Abingdon, England). 2010;23(1):355.

223. Farah LG, Fayyad JA, Eapen V, Cassir Y, Salamoun MM, Tabet CC, et al. ADHD in the Arab world: a review of epidemiologic studies. Journal of attention disorders. 2009;13(3):211-22.

224. Alhraiwil NJ, Ali A, Househ MS, Al-Shehri AM, El-Metwally AA. Systematic review of the epidemiology of attention deficit hyperactivity disorder in Arab countries. Neurosciences (Riyadh, Saudi Arabia). 2015;20(2):137-44.

225. Alkhateeb JM, Alhadidi MS. ADHD Research in Arab Countries: A Systematic Review of Literature. Journal of attention disorders. 2016;0.

226. Bakare MO, Munir KM. Autism spectrum disorders (ASD) in Africa: a perspective. African journal of psychiatry. 2011;14(3):208-10.

227. Salhia HO, Al-Nasser LA, Taher LS, Al-Khathaami AM, El-Metwally AA. Systemic review of the epidemiology of autism in Arab Gulf countries. Neurosciences (Riyadh, Saudi Arabia). 2014;19(4):291-6.

228. Esan O, Esan A. Epidemiology and burden of bipolar disorder in Africa: a systematic review of data from Africa. Soc Psychiatry Psychiatr Epidemiol. 2016;51(1):93-100.

229. Zahidie A, Jamali T. An overview of the predictors of depression among adult Pakistani women. Journal of the College of Physicians and Surgeons--Pakistan : JCPSP. 2013;23(8):574-80.

230. Travers KU, Pokora TD, Cadarette SM, Mould JF. Major depressive disorder in Africa and the Middle East: a systematic literature review. Expert review of pharmacoeconomics & outcomes research. 2013;13(5):613-30.

231. Dardas LA, Bailey DE, Jr., Simmons LA. Adolescent Depression in the Arab Region: A Systematic Literature Review. Issues Ment Health Nurs. 2016;37(8):569-85.

232. Klainin P, Arthur DG. Postpartum depression in Asian cultures: a literature review. Int J Nurs Stud. 2009;46(10):1355-73.

233. Cheng C, Li AY. Internet addiction prevalence and quality of (real) life: a meta-analysis of 31 nations across seven world regions. Cyberpsychology, behavior and social networking. 2014;17(12):755-60.

234. Nasser SC, Salamoun MM. Treatment of mental disorders and pathways to care in Arab countries. International journal of psychiatry in clinical practice. 2011;15(1):12-8.

235. Dimitry L. A systematic review on the mental health of children and adolescents in areas of armed conflict in the Middle East. Child Care Health Dev. 2012;38(2):153-61.

236. Kronfol Z, Saleh M, Al-Ghafry M. Mental health issues among migrant workers in Gulf Cooperation Council countries: literature review and case illustrations. Asian journal of psychiatry. 2014;10:109-13.

237. Marie M, Hannigan B, Jones A. Mental health needs and services in the West Bank, Palestine. International journal of mental health systems. 2016;10:23.

238. Hassan G, Ventevogel P, Jefee-Bahloul H, Barkil-Oteo A, Kirmayer LJ. Mental health and psychosocial wellbeing of Syrians affected by armed conflict. Epidemiology and psychiatric sciences. 2016;25(2):129-41.

239. Ayer L, Venkatesh B, Stewart R, Mandel D, Stein B, Schoenbaum M. Psychological Aspects of the Israeli-Palestinian Conflict: A Systematic Review. Trauma, violence & abuse. 2015;0.

240. Wells R, Steel Z, Abo-Hilal M, Hassan AH, Lawsin C. Psychosocial concerns reported by Syrian refugees living in Jordan: systematic review of unpublished needs assessments. The British journal of psychiatry : the journal of mental science. 2016;209(2):99-106.

241. Rezaeian M. Suicide among young Middle Eastern Muslim females. Crisis. 2010;31(1):36-42.

242. Morovatdar N, Moradi-Lakeh M, Malakouti SK, Nojomi M. Most common methods of suicide in Eastern Mediterranean Region of WHO: a systematic review and meta-analysis. Archives of suicide research : official journal of the International Academy for Suicide Research. 2013;17(4):335-44.

243. Jordans MJ, Kaufman A, Brenman NF, Adhikari RP, Luitel NP, Tol WA, et al. Suicide in South Asia: a scoping review. BMC Psychiatry. 2014;14:358.

244. Mars B, Burrows S, Hjelmeland H, Gunnell D. Suicidal behaviour across the African continent: a review of the literature. BMC Public Health. 2014;14:606.

245. Fuhr DC, Calvert C, Ronsmans C, Chandra PS, Sikander S, De Silva MJ, et al. Contribution of suicide and injuries to pregnancy-related mortality in low-income and middle-income countries: a systematic review and meta-analysis. Lancet Psychiatry. 2014;1(3):213-25.

246. Shahid M, Hyder AA. Deliberate self-harm and suicide: a review from Pakistan. Int J Inj Contr Saf Promot. 2008;15(4):233-41.

247. Al-Khateeb JM, Al-Khateeb AJ. Research on psychosocial aspects of epilepsy in Arab countries: a review of literature. Epilepsy & behavior : E&B. 2014;31:256-62.

248. Amawi N, Mollica RF, Lavelle J, Osman O, Nasir L. Overview of research on the mental health impact of violence in the Middle East in light of the Arab Spring. J Nerv Ment Dis. 2014;202(9):625-9.

249. van der Meer IM, Middelkoop BJ, Boeke AJ, Lips P. Prevalence of vitamin D deficiency among Turkish, Moroccan, Indian and sub-Sahara African populations in Europe and their countries of origin: an overview. Osteoporos Int. 2011;22(4):1009-21.

250. Badawi A, Arora P, Sadoun E, Al-Thani AA, Thani MH. Prevalence of vitamin d insufficiency in qatar: a systematic review. Journal of public health research. 2012;1(3):229-35.

251. Bassil D, Rahme M, Hoteit M, Fuleihan Gel H. Hypovitaminosis D in the Middle East and North Africa: Prevalence, risk factors and impact on outcomes. Dermatoendocrinol. 2013;5(2):274-98.

252. Hilger J, Friedel A, Herr R, Rausch T, Roos F, Wahl DA, et al. A systematic review of vitamin D status in populations worldwide. The British journal of nutrition. 2014;111(1):23-45.

253. Palacios C, Gonzalez L. Is vitamin D deficiency a major global public health problem? The Journal of steroid biochemistry and molecular biology. 2014;144:138-45.

254. Farrokhyar F, Tabasinejad R, Dao D, Peterson D, Ayeni OR, Hadioonzadeh R, et al. Prevalence of vitamin D inadequacy in athletes: a systematic-review and meta-analysis. Sports Med. 2015;45(3):365-78.

255. Al-Daghri NM. Vitamin D in Saudi Arabia: Prevalence,distribution and disease associations. The Journal of steroid biochemistry and molecular biology. 2016;0.

256. Agarwal A, Gupta SK, Sukumar R. Hyperparathyroidism and malnutrition with severe vitamin D deficiency. World journal of surgery. 2009;33(11):2303-13.

257. Mehboob B, Safdar NF, Zaheer S. Socio-economic, environmental and demographic determinants of rise in obesity among Pakistani women: A Systematic Review. JPMA The Journal of the Pakistan Medical Association. 2016;66(9):1165-72.

258. Hammad SS, Berry DC. The Child Obesity Epidemic in Saudi Arabia: A Review of the Literature. Journal of transcultural nursing : official journal of the Transcultural Nursing Society / Transcultural Nursing Society. 2016;0.

259. Danaei G, Finucane MM, Lin JK, Singh GM, Paciorek CJ, Cowan MJ, et al. National, regional, and global trends in systolic blood pressure since 1980: systematic analysis of health examination surveys and epidemiological studies with 786 country-years and 5.4 million participants. Lancet. 2011;377(9765):568-77.

260. Hasan DM, Emeash AH, Mustafa SB, Abdelazim GE, El-din AA. Hypertension in Egypt: a systematic review. Current hypertension reviews. 2014;10(3):134-41.

261. Neupane D, McLachlan CS, Sharma R, Gyawali B, Khanal V, Mishra SR, et al. Prevalence of hypertension in member countries of South Asian Association for Regional Cooperation (SAARC): systematic review and meta-analysis. Medicine (Baltimore). 2014;93(13):e74.

262. Tailakh A, Evangelista LS, Mentes JC, Pike NA, Phillips LR, Morisky DE. Hypertension prevalence, awareness, and control in Arab countries: a systematic review. Nursing & health sciences. 2014;16(1):126-30.

263. Nansseu JR, Noubiap JJ, Mengnjo MK, Aminde LN, Essouma M, Jingi AM, et al. The highly neglected burden of resistant hypertension in Africa: a systematic review and meta-analysis. BMJ Open. 2016;6(9):e011452.

264. Mabry RM, Reeves MM, Eakin EG, Owen N. Gender differences in prevalence of the metabolic syndrome in Gulf Cooperation Council Countries: a systematic review. Diabetic medicine : a journal of the British Diabetic Association. 2010;27(5):593-7.

265. Mirmiran P, Sherafat-Kazemzadeh R, Jalali-Farahani S, Azizi F. Childhood obesity in the Middle East: a review. Eastern Mediterranean health journal = La revue de sante de la Mediterranee orientale = al-Majallah al-sihhiyah li-sharq al-mutawassit. 2010;16(9):1009-17.

266. Karageorgi S, Alsmadi O, Behbehani K. A review of adult obesity prevalence, trends, risk factors, and epidemiologic methods in Kuwait. J Obes. 2013;2013:378650.

267. Toselli S, Gualdi-Russo E, Boulos DN, Anwar WA, Lakhoua C, Jaouadi I, et al. Prevalence of overweight and obesity in adults from North Africa. Eur J Public Health. 2014;24:31-9.

268. Mistry SK, Puthussery S. Risk factors of overweight and obesity in childhood and adolescence in South Asian countries: a systematic review of the evidence. Public Health. 2015;129(3):200-9.

269. Alhyas L, McKay A, Balasanthiran A, Majeed A. Prevalences of overweight, obesity, hyperglycaemia, hypertension and dyslipidaemia in the Gulf: systematic review. JRSM short reports. 2011;2(7):55.

270. Farzadfar F, Finucane MM, Danaei G, Pelizzari PM, Cowan MJ, Paciorek CJ, et al. National, regional, and global trends in serum total cholesterol since 1980: systematic analysis of health examination surveys and epidemiological studies with 321 country-years and 3.0 million participants. Lancet. 2011;377(9765):578-86.

271. Bamimore MA, Zaid A, Banerjee Y, Al-Sarraf A, Abifadel M, Seidah NG, et al. Familial hypercholesterolemia mutations in the Middle Eastern and North African region: a need for a national registry. Journal of clinical lipidology. 2015;9(2):187-94.

272. Abou Abbas L, Salameh P, Nasser W, Nasser Z, Godin I. Obesity and symptoms of depression among adults in selected countries of the Middle East: a systematic review and meta-analysis. Clinical obesity. 2015;5(1):2-11.

273. Sadat-Ali M, Al-Habdan IM, Al-Turki HA, Azam MQ. An epidemiological analysis of the incidence of osteoporosis and osteoporosis-related fractures among the Saudi Arabian population. Ann Saudi Med. 2012;32(6):637-41.

274. Baddoura R, Hoteit M, El-Hajj Fuleihan G. Osteoporotic fractures, DXA, and fracture risk assessment: meeting future challenges in the Eastern Mediterranean Region. Journal of clinical densitometry : the official journal of the International Society for Clinical Densitometry. 2011;14(4):384-94.

275. Najjar H, Easson A. Age at diagnosis of breast cancer in Arab nations. International journal of surgery (London, England). 2010;8(6):448-52.

276. Bhikoo R, Srinivasa S, Yu TC, Moss D, Hill AG. Systematic review of breast cancer biology in developing countries (part 1): Africa, the middle East, eastern europe, Mexico, the Caribbean and South america. Cancers. 2011;3(2):2358-81.

277. Alhurishi S, Lim JN, Potrata B, West R. Factors influencing late presentation for breast cancer in the middle East: a systematic review. Asian Pacific journal of cancer prevention : APJCP. 2011;12(6):1597-600.

278. Corbex M, Bouzbid S, Boffetta P. Features of breast cancer in developing countries, examples from North-Africa. European journal of cancer (Oxford, England : 1990). 2014;50(10):1808-18.

279. Dubey AK, Gupta U, Jain S. Breast cancer statistics and prediction methodology: a systematic review and analysis. Asian Pacific journal of cancer prevention : APJCP. 2015;16(10):4237-45.

280. Ting J, Kruzikas DT, Smith JS. A global review of age-specific and overall prevalence of cervical lesions. International journal of gynecological cancer : official journal of the International Gynecological Cancer Society. 2010;20(7):1244-9.

281. Alhazzazi TY, Alghamdi FT. Head and Neck Cancer in Saudi Arabia: a Systematic Review. Asian Pacific journal of cancer prevention : APJCP. 2016;17(8):4043-8.

282. Ashtari S, Pourhoseingholi MA, Sharifian A, Zali MR. Hepatocellular carcinoma in Asia: Prevention strategy and planning. World journal of hepatology. 2015;7(12):1708-17.

283. Dubey AK, Gupta U, Jain S. Epidemiology of lung cancer and approaches for its prediction: a systematic review and analysis. Chinese journal of cancer. 2016;35(1):71.

284. Ahmed HG. Aetiology of oral cancer in the Sudan. J Oral Maxillofac Res. 2013;4(2):e3.

285. Krishna Rao SV, Mejia G, Roberts-Thomson K, Logan R. Epidemiology of oral cancer in Asia in the past decade--an update (2000-2012). Asian Pacific journal of cancer prevention : APJCP. 2013;14(10):5567-77.

286. BenNasir E, El Mistiri M, McGowan R, Katz RV. Oral cancer in Libya and development of regional oral cancer registries: A review. The Saudi dental journal. 2015;27(4):171-9.

287. Al-Jaber A, Al-Nasser L, El-Metwally A. Epidemiology of oral cancer in Arab countries. Saudi Med J. 2016;37(3):249-55.

288. Sung PL, Chang YH, Chao KC, Chuang CM. Global distribution pattern of histological subtypes of epithelial ovarian cancer: a database analysis and systematic review. Gynecologic oncology. 2014;133(2):147-54.

289. Cherbal F, Bakour R, Adane S, Boualga K. BRCA1 and BRCA2 germline mutation spectrum in hereditary breast/ovarian cancer families from Maghrebian countries. Breast Dis. 2012;34(1):1-8.

290. Laraqui A, Uhrhammer N, Rhaffouli HE, Sekhsokh Y, Lahlou-Amine I, Bajjou T, et al. BRCA genetic screening in Middle Eastern and North African: mutational spectrum and founder BRCA1 mutation (c.798_799delTT) in North African. Dis Markers. 2015;2015:194293.

291. Oluwagbemiga LA, Oluwole A, Kayode AA. Seventeen years after BRCA1: what is the BRCA mutation status of the breast cancer patients in Africa? - a systematic review. SpringerPlus. 2012;1(1):83.

292. Eng A, McCormack V, dos-Santos-Silva I. Receptor-defined subtypes of breast cancer in indigenous populations in Africa: a systematic review and meta-analysis. PLoS medicine. 2014;11(9):e1001720.

293. Midha A, Dearden S, McCormack R. EGFR mutation incidence in non-small-cell lung cancer of adenocarcinoma histology: a systematic review and global map by ethnicity (mutMapII). American journal of cancer research. 2015;5(9):2892-911.

294. Irshad M, Mandal RK, Al-Drees A, Khalil MS, Abdulghani HM. No Evidence of Association of the Arg72Pro p53 Gene Polymorphism with Cancer Risk in the Saudi Population: a Meta-Analysis. Asian Pacific journal of cancer prevention : APJCP. 2015;16(14):5663-7.

295. Shaik AP, Shaik AS, Al-Sheikh YA. Colorectal cancer: A review of the genome-wide association studies in the kingdom of Saudi Arabia. Saudi J Gastroenterol. 2015;21(3):123-8.

296. Huang H, Hu XF, Zhao FH, Garland SM, Bhatla N, Qiao YL. Estimation of Cancer Burden Attributable to Infection in Asia. Journal of epidemiology / Japan Epidemiological Association. 2015;25(10):626-38.

297. Hussein WM, Anwar WA, Attaleb M, Mazini L, Forsti A, Trimbitas RD, et al. A review of the infection-associated cancers in North African countries. Infectious agents and cancer. 2016;11:35.

298. Baandrup L, Thomsen LT, Olesen TB, Andersen KK, Norrild B, Kjaer SK. The prevalence of human papillomavirus in colorectal adenomas and adenocarcinomas: a systematic review and meta-analysis. European journal of cancer (Oxford, England : 1990). 2014;50(8):1446-61.

299. Haddou Rahou B, El Rhazi K, Ouasmani F, Nejjari C, Bekkali R, Montazeri A, et al. Quality of life in Arab women with breast cancer: a review of the literature. Health and quality of life outcomes. 2016;14:64.

300. Donnelly TT, Khater AH, Al-Bader SB, Al Kuwari MG, Al-Meer N, Malik M, et al. Arab women's breast cancer screening practices: a literature review. Asian Pacific journal of cancer prevention : APJCP. 2013;14(8):4519-28.

301. Alananzeh I, Levesque J, Kwok C, Everett B. Integrative Review of the Supportive Care Needs of Arab People Affected by Cancer. Asia-Pacific journal of oncology nursing. 2016;3(2):148-56.

302. Peleteiro B, Bastos J, Barros H, Lunet N. Systematic review of the prevalence of gastric intestinal metaplasia and its area-level association with smoking. Gaceta sanitaria / SESPAS. 2008;22(3):236-47; discussion 46-7.

303. Almaguer M, Herrera R, Orantes CM. Chronic kidney disease of unknown etiology in agricultural communities. MEDICC review. 2014;16(2):9-15.

304. Hassanien AA, Al-Shaikh F, Vamos EP, Yadegarfar G, Majeed A. Epidemiology of end-stage renal disease in the countries of the Gulf Cooperation Council: a systematic review. JRSM short reports. 2012;3(6):38.

305. Goleg FA, Kong NC, Sahathevan R. Dialysis-treated end-stage kidney disease in Libya: epidemiology and risk factors. International urology and nephrology. 2014;46(8):1581-7.

306. Okpechi IG, Ameh OI, Bello AK, Ronco P, Swanepoel CR, Kengne AP. Epidemiology of Histologically Proven Glomerulonephritis in Africa: A Systematic Review and Meta-Analysis. PLoS One. 2016;11(3):e0152203.

307. McGrogan A, Franssen CF, de Vries CS. The incidence of primary glomerulonephritis worldwide: a systematic review of the literature. Nephrology, dialysis, transplantation : official publication of the European Dialysis and Transplant Association - European Renal Association. 2011;26(2):414-30.

308. Faust WC, Diaz M, Pohl HG. Incidence of post-pyelonephritic renal scarring: a meta-analysis of the dimercapto-succinic acid literature. The Journal of urology. 2009;181(1):290-7; discussion 7-8.

309. Gwer S, Chacha C, Newton CR, Idro R. Childhood acute non-traumatic coma: aetiology and challenges in management in resource-poor countries of Africa and Asia. Paediatrics and international child health. 2013;33(3):129-38.

310. Marin B, Boumediene F, Logroscino G, Couratier P, Babron MC, Leutenegger AL, et al. Variation in worldwide incidence of amyotrophic lateral sclerosis: a meta-analysis. International journal of epidemiology. 2016;0.

311. Benamer HT, Grosset DG. A systematic review of the epidemiology of epilepsy in Arab countries. Epilepsia. 2009;50(10):2301-4.

312. Angalakuditi M, Angalakuditi N. A comprehensive review of the literature on epilepsy in selected countries in emerging markets. Neuropsychiatric disease and treatment. 2011;7:585-97.

313. Bhalla D, Lotfalinezhad E, Timalsina U, Kapoor S, Kumar KS, Abdelrahman A, et al. A comprehensive review of epilepsy in the Arab world. Seizure. 2016;34:54-9.

314. Benamer HT, Bredan A. Guillain-Barre syndrome in Arab countries: a systematic review. J Neurol Sci. 2014;343(1):221-3.

315. Webb AJ, Brain SA, Wood R, Rinaldi S, Turner MR. Seasonal variation in Guillain-Barre syndrome: a systematic review, meta-analysis and Oxfordshire cohort study. Journal of neurology, neurosurgery, and psychiatry. 2015;86(11):1196-201.

316. Benamer HT, Deleu D, Grosset D. Epidemiology of headache in Arab countries. J Headache Pain. 2010;11(1):1-3.

317. Al-Hashel J, Besterman AD, Wolfson C. The prevalence of multiple sclerosis in the Middle East. Neuroepidemiology. 2008;31(2):129-37.

318. Heydarpour P, Khoshkish S, Abtahi S, Moradi-Lakeh M, Sahraian MA. Multiple Sclerosis Epidemiology in Middle East and North Africa: A Systematic Review and Meta-Analysis. Neuroepidemiology. 2015;44(4):232-44.

319. Eskandarieh S, Heydarpour P, Minagar A, Pourmand S, Sahraian MA. Multiple Sclerosis Epidemiology in East Asia, South East Asia and South Asia: A Systematic Review. Neuroepidemiology. 2016;46(3):209-21.

320. Benamer HT, Ahmed ES, Al-Din AS, Grosset DG. Frequency and clinical patterns of multiple sclerosis in Arab countries: a systematic review. J Neurol Sci. 2009;278(1):1-4.

321. Benamer HT, de Silva R, Siddiqui KA, Grosset DG. Parkinson's disease in Arabs: a systematic review. Movement disorders : official journal of the Movement Disorder Society. 2008;23(9):1205-10.

322. Benamer HT, Bredan A. The epidemiology of myasthenia gravis in Arab countries: a systematic review. Muscle & nerve. 2015;51(1):144-5.

323. Correia Guedes L, Ferreira JJ, Rosa MM, Coelho M, Bonifati V, Sampaio C. Worldwide frequency of G2019S LRRK2 mutation in Parkinson's disease: a systematic review. Parkinsonism Relat Disord. 2010;16(4):237-42.

324. Amara AH, Aljunid SM. Noncommunicable diseases among urban refugees and asylum-seekers in developing countries: a neglected health care need. Globalization and health. 2014;10:24.

325. Al-Qasem A, Smith F, Clifford S. Adherence to medication among chronic patients in Middle Eastern countries: review of studies. Eastern Mediterranean health journal = La revue de sante de la Mediterranee orientale = al-Majallah al-sihhiyah li-sharq al-mutawassit. 2011;17(4):356-63.

326. Ng SW, Zaghloul S, Ali HI, Harrison G, Popkin BM. The prevalence and trends of overweight, obesity and nutrition-related non-communicable diseases in the Arabian Gulf States. Obes Rev. 2011;12(1):1-13.

327. Boutayeb A, Boutayeb S, Boutayeb W. Multi-morbidity of non communicable diseases and equity in WHO Eastern Mediterranean countries. Int J Equity Health. 2013;12:60.

328. John LJ, Shantakumari N. Herbal Medicines Use During Pregnancy: A Review from the Middle East. Oman medical journal. 2015;30(4):229-36.

329. Nazeri P, Mirmiran P, Shiva N, Mehrabi Y, Mojarrad M, Azizi F. Iodine nutrition status in lactating mothers residing in countries with mandatory and voluntary iodine fortification programs: an updated systematic review. Thyroid. 2015;25(6):611-20.

330. Mirmiran P, Golzarand M, Serra-Majem L, Azizi F. Iron, iodine and vitamin a in the middle East; a systematic review of deficiency and food fortification. Iranian journal of public health. 2012;41(8):8-19.

331. Nielsen J, Prudhon C, de Radigues X. Trends in malnutrition and mortality in Darfur, Sudan, between 2004 and 2008: a meta-analysis of publicly available surveys. International journal of epidemiology. 2011;40(4):971-84.

332. Tsigga M, Grammatikopoulou MG. Assessing the silent epidemic of malnutrition in Palestinian preschool children. Journal of epidemiology and global health. 2012;2(4):181-91.

333. Akhtar S. Malnutrition in South Asia-A Critical Reappraisal. Critical reviews in food science and nutrition. 2016;56(14):2320-30.

334. Musaiger AO, Hassan AS, Obeid O. The paradox of nutrition-related diseases in the Arab countries: the need for action. International journal of environmental research and public health. 2011;8(9):3637-71.

335. Hirani SA. Malnutrition in young Pakistani children. Journal of Ayub Medical College, Abbottabad : JAMC. 2012;24(2):150-3.

336. Best C, Neufingerl N, van Geel L, van den Briel T, Osendarp S. The nutritional status of school-aged children: why should we care? Food and nutrition bulletin. 2010;31(3):400-17.

337. Stark KD, Van Elswyk ME, Higgins MR, Weatherford CA, Salem N, Jr. Global survey of the omega-3 fatty acids, docosahexaenoic acid and eicosapentaenoic acid in the blood stream of healthy adults. Progress in lipid research. 2016;63:132-52.

338. Creo AL, Thacher TD, Pettifor JM, Strand MA, Fischer PR. Nutritional rickets around the world: an update. Paediatrics and international child health. 2016;0:1-15.

339. Stoffaneller R, Morse NL. A review of dietary selenium intake and selenium status in Europe and the Middle East. Nutrients. 2015;7(3):1494-537.

340. Powles J, Fahimi S, Micha R, Khatibzadeh S, Shi P, Ezzati M, et al. Global, regional and national sodium intakes in 1990 and 2010: a systematic analysis of 24 h urinary sodium excretion and dietary surveys worldwide. BMJ Open. 2013;3(12):e003733.

341. Sharma IK, Byrne A. Early initiation of breastfeeding: a systematic literature review of factors and barriers in South Asia. International breastfeeding journal. 2016;11:17.

342. Ahmad K, A BZ, D JMT, Chand B. A systematic review of epidemiological literature on the eye health of marginalized fishing populations. JPMA The Journal of the Pakistan Medical Association. 2016;66(10):S81-s3.

343. Stevens GA, White RA, Flaxman SR, Price H, Jonas JB, Keeffe J, et al. Global prevalence of vision impairment and blindness: magnitude and temporal trends, 1990-2010. Ophthalmology. 2013;120(12):2377-84.

344. Bourne R, Price H, Taylor H, Leasher J, Keeffe J, Glanville J, et al. New systematic review methodology for visual impairment and blindness for the 2010 Global Burden of Disease study. Ophthalmic epidemiology. 2013;20(1):33-9.

345. Cheng JW, Zong Y, Zeng YY, Wei RL. The prevalence of primary angle closure glaucoma in adult Asians: a systematic review and meta-analysis. PLoS One. 2014;9(7):e103222.

346. Khairallah M, Kahloun R, Flaxman SR, Jonas JB, Keeffe J, Leasher J, et al. Prevalence and causes of vision loss in North Africa and the Middle East: 1990-2010. The British journal of ophthalmology. 2014;98(5):605-11.

347. Gilbert CE, Lepvrier-Chomette N. Gender Inequalities in Surgery for Bilateral Cataract among Children in Low-Income Countries: A Systematic Review. Ophthalmology. 2016;123(6):1245-51.

348. Khan SQ, Khan NB, Arrejaie AS. Dental caries. A meta analysis on a Saudi population. Saudi Med J. 2013;34(7):744-9.

349. Al-Bluwi GS. Epidemiology of dental caries in children in the United Arab Emirates. International dental journal. 2014;64(4):219-28.

350. Khan SQ. Dental caries in Arab League countries: a systematic review and meta-analysis. International dental journal. 2014;64(4):173-80.

351. Al Agili DE. A systematic review of population-based dental caries studies among children in Saudi Arabia. The Saudi dental journal. 2013;25(1):3-11.

352. Barzangi J, Unell L, Soderfeldt B, Arnrup K. Infant dental enucleation: A literature review on a traditional remedial practice in East Africa. Acta odontologica Scandinavica. 2014;72(3):168-78.

353. Al-Nasser L, El-Metwally A. Oral lichen planus in Arab countries : a review. Journal of oral pathology & medicine : official publication of the International Association of Oral Pathologists and the American Academy of Oral Pathology. 2014;43(10):723-7.

354. Al-Harthi LS, Cullinan MP, Leichter JW, Thomson WM. Periodontitis among adult populations in the Arab World. International dental journal. 2013;63(1):7-11.

355. Mohamed Zaki LR, Hairi NN. A Systematic Review of the Prevalence and Measurement of Chronic Pain in Asian Adults. Pain management nursing : official journal of the American Society of Pain Management Nurses. 2015;16(3):440-52.

356. Sisson SB, Katzmarzyk PT. International prevalence of physical activity in youth and adults. Obes Rev. 2008;9(6):606-14.

357. Mabry RM, Reeves MM, Eakin EG, Owen N. Evidence of physical activity participation among men and women in the countries of the Gulf cooperation council: a review. Obes Rev. 2010;11(6):457-64.

358. Ranasinghe CD, Ranasinghe P, Jayawardena R, Misra A. Physical activity patterns among South-Asian adults: a systematic review. Int J Behav Nutr Phys Act. 2013;10:116.

359. Yammine K. The prevalence of physical activity among the young population of UAE: a meta-analysis. Perspectives in public health. 2016;0.

360. Mabry R, Koohsari MJ, Bull F, Owen N. A systematic review of physical activity and sedentary behaviour research in the oil-producing countries of the Arabian Peninsula. BMC Public Health. 2016;16(1):1003.

361. Loney T, Aw TC, Handysides DG, Ali R, Blair I, Grivna M, et al. An analysis of the health status of the United Arab Emirates: the 'Big 4' public health issues. Glob Health Action. 2013;6:20100.

362. Adeloye D, Chua S, Lee C, Basquill C, Papana A, Theodoratou E, et al. Global and regional estimates of COPD prevalence: Systematic review and meta-analysis. Journal of global health. 2015;5(2):020415.

363. Akhter E, Bilal S, Kiani A, Haque U. Prevalence of arthritis in India and Pakistan: a review. Rheumatology international. 2011;31(7):849-55.

364. Usenbo A, Kramer V, Young T, Musekiwa A. Prevalence of Arthritis in Africa: A Systematic Review and Meta-Analysis. PLoS One. 2015;10(8):e0133858.

365. Almoallim HM, Alharbi LA. Rheumatoid arthritis in Saudi Arabia. Saudi Med J. 2014;35(12):1442-54.

366. Stolwijk C, van Onna M, Boonen A, van Tubergen A. Global Prevalence of Spondyloarthritis: A Systematic Review and Meta-Regression Analysis. Arthritis Care Res (Hoboken). 2016;68(9):1320-31.

367. Mustafa KN. Takayasu's arteritis in Arabs. Clinical rheumatology. 2014;33(12):1777-83.

368. Osio-Salido E, Manapat-Reyes H. Epidemiology of systemic lupus erythematosus in Asia. Lupus. 2010;19(12):1365-73.

369. Rafeey M, Ghojazadeh M, Mehdizadeh A, Hazrati H, Vahedi L. Intercontinental comparison of caustic ingestion in children. Korean J Pediatr. 2015;58(12):491-500.

370. Kadir MM, Janjua NZ, Kristensen S, Fatmi Z, Sathiakumar N. Status of children's blood lead levels in Pakistan: implications for research and policy. Public Health. 2008;122(7):708-15.

371. Chippaux JP, Goyffon M. Epidemiology of scorpionism: a global appraisal. Acta tropica. 2008;107(2):71-9.

372. Othman N, Kendrick D. Epidemiology of burn injuries in the East Mediterranean Region: a systematic review. BMC Public Health. 2010;10:83.

373. Golshan A, Patel C, Hyder AA. A systematic review of the epidemiology of unintentional burn injuries in South Asia. J Public Health (Oxf). 2013;35(3):384-96.

374. Tapp C, Burkle FM, Jr., Wilson K, Takaro T, Guyatt GH, Amad H, et al. Iraq War mortality estimates: a systematic review. Conflict and health. 2008;2:1.

375. Fehling M, Jarrah ZM, Tiernan ME, Albezreh S, VanRooyen MJ, Alhokair A, et al. Youth in crisis in the Middle East and North Africa: a systematic literature review and focused landscape analysis. Eastern Mediterranean health journal = La revue de sante de la Mediterranee orientale = al-Majallah al-sihhiyah li-sharq al-mutawassit. 2016;21(12):916-30.

376. Cheng SY, Levy AR, Lefaivre KA, Guy P, Kuramoto L, Sobolev B. Geographic trends in incidence of hip fractures: a comprehensive literature review. Osteoporos Int. 2011;22(10):2575-86.

377. Kulczycki A, Windle S. Honor killings in the Middle East and North Africa: a systematic review of the literature. Violence Against Women. 2011;17(11):1442-64.

378. Ali PA, Naylor PB, Croot E, O'Cathain A. Intimate Partner Violence in Pakistan: A Systematic Review. Trauma, violence & abuse. 2015;16(3):299-315.

379. Roman NV, Frantz JM. The prevalence of intimate partner violence in the family: a systematic review of the implications for adolescents in Africa. Fam Pract. 2013;30(3):256-65.

380. Boy A, Kulczycki A. What we know about intimate partner violence in the Middle East and North Africa. Violence Against Women. 2008;14(1):53-70.

381. Majzoub AA, Canguven O, Raidh TA. Alteration in the etiology of penile fracture in the Middle East and Central Asia regions in the last decade; a literature review. Urol Ann. 2015;7(3):284-8.

382. McAlpine A, Hossain M, Zimmerman C. Sex trafficking and sexual exploitation in settings affected by armed conflicts in Africa, Asia and the Middle East: systematic review. BMC Int Health Hum Rights. 2016;16(1):34.

383. Mansuri FA, Al-Zalabani AH, Zalat MM, Qabshawi RI. Road safety and road traffic accidents in Saudi Arabia. A systematic review of existing evidence. Saudi Med J. 2015;36(4):418-24.

384. Puvanachandra P, Hoe C, El-Sayed HF, Saad R, Al-Gasseer N, Bakr M, et al. Road traffic injuries and data systems in Egypt: addressing the challenges. Traffic Inj Prev. 2012;13:44-56.

385. Abou-Abbass H, Bahmad H, Ghandour H, Fares J, Wazzi-Mkahal R, Yacoub B, et al. Epidemiology and clinical characteristics of traumatic brain injury in Lebanon: A systematic review. Medicine (Baltimore). 2016;95(47):e5342.

386. Cripps RA, Lee BB, Wing P, Weerts E, Mackay J, Brown D. A global map for traumatic spinal cord injury epidemiology: towards a living data repository for injury prevention. SPINAL CORD. 2011;49(4):493-501.

387. Hillis S, Mercy J, Amobi A, Kress H. Global Prevalence of Past-year Violence Against Children: A Systematic Review and Minimum Estimates. Pediatrics. 2016;137(3):e20154079.
