# Supplementary material for: Strengthening literature search strategies for systematic reviews reporting population health in the Middle East and North Africa: A meta‐research study
Source: J Evid Based Med. 2020 May 24;13(3):192–8. doi: 10.1111/jebm.12394 (PMC7497175; doi:10.1111/jebm.12394)
Supplement: Supplementary file 3 — Supporting Information [file JEBM-13-192-s003.docx]

List of commercial sources of literature searched by the systematic reviews reporting population health data in MENA

| **Publisher/compagnies** |  |
| --- | --- |
|  | Cochrane |
|  | EBSCO |
|  | Elsevier |
|  | HighWire Press |
|  | Joanna Briggs Institute (JBI) |
|  | LexisNexis |
|  | ProQuest |
|  | Springer Science+Business Media |
|  | Taylor & Francis |
|  | Wiley-Blackwell |
| **Digital Libraries** |  |
|  | Abstracts in Social Gerontology – EBSCO |
|  | Academic OneFile – GALE |
|  | Academic Search Complete – EBSCO |
|  | Africa-Wife Information (formerly Africa-Wide NIPAD) – EBSCO |
|  | African HealthLine – Africa-Wife Information |
|  | African Index Medicus (AIM) – World Health Organization (WHO) |
|  | Allied and Complementary Medicine Database (AMED) – EBSCO or Ovid |
|  | AnthroSource – American Anthropology Association (AAA) – (Wiley Online Library) |
|  | Applied Social Sciences Index and Abstracts (ASSIA) – ProQuest |
|  | ArticleFirst – Online Computer Library Centre (OCLC) |
|  | Association for Computing Machinery (AMC) Digitial Library (DL) |
|  | Australian Sport Database (AUSPORT) – Australian governmental database but requires library subscription to access |
|  | Biological Abstracts – EBSCO or Ovid |
|  | BioMed Central (BMC) |
|  | BIOSIS Previews – EBSCO or Ovid |
|  | British Nursing Index – ProQuest |
|  | CAB Abstracts – CABI (non-profit organization, but requires subscription to access |
|  | Cochrane Central Register of Controlled Trials (CENTRAL) |
|  | Cochrane Collection Plus (EBSCO) |
|  | Cochrane Database of Systematic Reviews (CDSR) |
|  | ComDisDome - ProQuest |
|  | Contemporary Women's Issues (CWI) – GALE |
|  | COS Conference Papers Index – ProQuest |
|  | Cumulative Index of Nursing and Allied Health (CINAHL) |
|  | Cumulative Index of Nursing and Allied Health (CINAHL) Plus – EBSCO |
|  | Current Contents – Ovid |
|  | Dentistry and Oral Sciences Source (DOSS) - EBSCO |
|  | Dissertation Abstracts International – ProQuest |
|  | Dissertations & Theses Global – ProQuest |
|  | E-Journals Database – EBSCO |
|  | EBSCO Discovery Service |
|  | EBSCO GreenFILE (Free Environmental Database) |
|  | EBSCOHost |
|  | EBSCO Medline |
|  | Education Resources Information Center (ERIC) |
|  | Elsevier Masson (EM) Consulte Database |
|  | Elsevier Masson (EM) Premium |
|  | EMABSE – Elsevier |
|  | EMABSE Classic - Elsevier |
|  | Global Health - CABI |
|  | Index Medicus (IM) – MEDLINE |
|  | Institute for Scientific Information (ISI) Web of Science – formerly known as Web of Knowledge |
|  | International Bibliography of the Social Sciences (IBSS) |
|  | International Pharmaceutical Abstracts (IPA) – EBSCO or Ovid or ProQuest |
|  | JBI Databases |
|  | Journals (Oxford Academic) – Oxford University Press |
|  | JSTOR |
|  | LexisNexis Academic |
|  | Library, Information Science & Technology Abstracts (EBSCO LISTA) |
|  | MD Consult (changing to ClincalKey) – Elsevier |
|  | MEDLINE |
|  | Medscape |
|  | Middle Eastern & Central Asian Studies – EBSCO |
|  | National Electronic Library for Health (NeLH) |
|  | NIH CRISP Database (now part of NIH RePORT) |
|  | Ovid MEDLINE |
|  | Ovid – Wolters Kluwer |
|  | Oxford Scholarship Online – Oxford University Press |
|  | PEMSoft - EBSCO |
|  | Pharmacogenomics Knowledge Base (PharmGKB) |
|  | Population Information Online (POPLINE) |
|  | ProQuest Central |
|  | ProQuest Dissertations & Theses |
|  | ProQuest Dissertations & Theses A&I: Health & Medicine |
|  | ProQuest Dissertations & Theses A&I: Social Sciences |
|  | Psychology and Behavioural Sciences Collection – EBSCO |
|  | PsycINFO |
|  | PsycNET |
|  | Public Health Database – ProQuest |
|  | Public Health Genomics Knowledge Base (PHGKB ) – CDC |
|  | Science Citation Index Expanded (SCIE) |
|  | ScienceDirect - Elsevier |
|  | Scientific Electronic Library Online (SciELO) – BIREME |
|  | SciSearch – ProQuest |
|  | SCOPUS – ScienceDirect |
|  | Sciences Citation Index (SCI) |
|  | Social Sciences Citation Index (SSCI) |
|  | Social Services Abstracts - ProQuest |
|  | Sociological Abstracts - ProQuest |
|  | Sociology Research Database (SocINDEX) – EBSCO |
|  | SPORTSDiscus – EBSCO |
|  | SpringerLink |
|  | Studies on Women and Gender Abstracts (SWGA) – Taylor and Francis |
|  | Taylor and Francis Online |
|  | Violence Against Women – World Health Organization (WHO) |
|  | Women’s Studies International – EBSCO |
|  | WorldCat |
|  | Zetoc |
|  | Zoological Record – Web of Science |
| **Journals** |  |
|  | Academic Medicine |
|  | Advances in Health Sciences Education |
|  | Annals of Saudi Medicine |
|  | Arab Journal of Psychiatry |
|  | Asia-Pacific Population Journal |
|  | Avicenna Journal of Medicine |
|  | Bahrain Medical Bulletin |
|  | Clinical Evidence (BMJ) |
|  | Deutsches Ärzteblatt International |
|  | Eastern Mediterranean Health Journal |
|  | International Journal of Diabetes and Metabolism |
|  | Jordan Medical Journal |
|  | Kuwait Medical Journal |
|  | Medical Education |
|  | Medical Education Online |
|  | Medical Teacher |
|  | Middle East Journal of Age and Ageing |
|  | Middle Eastern Studies |
|  | Oman Medical Journal |
|  | Qatar Medical Journal |
|  | Saudi Dental Journal |
|  | Saudi Epidemiological Journal (indexed in PubMed) |
|  | Saudi Medical Journal |
|  | Sudan Medical Journal |
|  | Teaching and Learning in Medicine |
|  | The BMJ |
|  | The Lancet |
|  | Yemeni Journal for Medical Sciences |
| **Search Engines** |  |
|  | Bing |
|  | Google |
|  | Google Scholar |
|  | PubMed |
|  | Scirus (retired) – Elsevier |
|  | The Summon^®^ Service - ProQuest |
|  | Yahoo |
| **Other** |  |
|  | Global Burden of Disease |
|  | NetScape |
